# Promoter Architecture and Transcriptional Regulation of Genes Upregulated in Germination and Coleoptile Elongation of Diverse Rice Genotypes Tolerant to Submergence

**DOI:** 10.3389/fgene.2021.639654

**Published:** 2021-03-16

**Authors:** Bijayalaxmi Mohanty

**Affiliations:** NUS Environmental Research Institute, National University of Singapore, Singapore, Singapore

**Keywords:** rice, germination and coleoptile elongation, submergence tolerance, promoter *cis*-element, transcription factor, phytohormones, plant breeding

## Abstract

Rice has the natural morphological adaptation to germinate and elongate its coleoptile under submerged flooding conditions. The phenotypic deviation associated with the tolerance to submergence at the germination stage could be due to natural variation. However, the molecular basis of this variation is still largely unknown. A comprehensive understanding of gene regulation of different genotypes that have diverse rates of coleoptile elongation can provide significant insights into improved rice varieties. To do so, publicly available transcriptome data of five rice genotypes, which have different lengths of coleoptile elongation under submergence tolerance, were analyzed. The aim was to identify the correlation between promoter architecture, associated with transcriptional and hormonal regulation, in diverse genotype groups of rice that have different rates of coleoptile elongation. This was achieved by identifying the putative *cis*-elements present in the promoter sequences of genes upregulated in each group of genotypes (tolerant, highly tolerant, and extremely tolerant genotypes). Promoter analysis identified transcription factors (TFs) that are common and unique to each group of genotypes. The candidate TFs that are common in all genotypes are MYB, bZIP, AP2/ERF, ARF, WRKY, ZnF, MADS-box, NAC, AS2, DOF, E2F, ARR-B, and HSF. However, the highly tolerant genotypes interestingly possess binding sites associated with HY5 (bZIP), GBF3, GBF4 and GBF5 (bZIP), DPBF-3 (bZIP), ABF2, ABI5, bHLH, and BES/BZR, in addition to the common TFs. Besides, the extremely tolerant genotypes possess binding sites associated with bHLH TFs such as BEE2, BIM1, BIM3, BM8 and BAM8, and ABF1, in addition to the TFs identified in the tolerant and highly tolerant genotypes. The transcriptional regulation of these TFs could be linked to phenotypic variation in coleoptile elongation in response to submergence tolerance. Moreover, the results indicate a cross-talk between the key TFs and phytohormones such as gibberellic acid, abscisic acid, ethylene, auxin, jasmonic acid, and brassinosteroids, for an altered transcriptional regulation leading to differences in germination and coleoptile elongation under submergence. The information derived from the current *in silico* analysis can potentially assist in developing new rice breeding targets for direct seeding.

## Introduction

Rice is one of the major cereal crops and staple food in Southeast Asian countries. In most of these countries, people sow seeds in the flooded rice fields through direct seeding that helps in reducing cost and manpower. During this process, rice seeds encounter flooding/submergence stress as they are exposed to hypoxia and even anoxia. Rice has the natural morphological adaptation to germinate and elongate its coleoptile under submerged flooding condition and also even in anoxic conditions. This coleoptile reaches the surface of water to get O_2_ for aerobic respiration to get sufficient energy for the development of roots and shoots (Narsai et al., 2015). However, there is phenotypic variation in coleoptile elongation and the tolerance to submergence at the germination stage among different genotypes and which could be due to natural variation. Tolerant genotypes germinate and elongate their coleoptile and hydrolyze starch to sugars for glycolysis and enhanced ethanolic fermentation to generate ATP (Lee et al., 2009). Besides, the plant hormone ethylene plays a major role during this adaptation (Ma et al., 2010; Steffens et al., 2012). Although a number of genetic studies provide information that the extent of tolerance to flooding in rice at the germination stage is due to natural variation (Septiningsih et al., 2013; Baltazar et al., 2014; Hsu and Tung, 2015), the molecular mechanism behind this phenotypic variation is largely unknown. A comprehensive understanding of the gene regulation of different genotypes tolerant to submergence/flooding can provide a significant insight into breeding of improved tolerant rice varieties for direct seeding cultivation.

A number of microarray and RNA-sequencing studies performed on rice germination under anoxia/hypoxia have shown the up-/downregulation of a number of stress-responsive genes (Lasanthi-Kudahettige et al., 2007; Narsai et al., 2009; Shingaki-Wells et al., 2011; Narsai et al., 2015; Hsu and Tung, 2017; Wu and Yang, 2020). During such stress conditions, transcription factors (TFs) play a key role in regulating the expression of genes that support them to survive through tolerance mechanism. The molecular mechanism of submergence has been studied on the rice genotype FR13A, which is able to survive 2 weeks as it has *Submergence 1* (*SUB1*) locus that encodes genes such as *SUB1A*, *SUB1B*, and *SUB1C* that belong to the ethylene-response factor (ERF) subgroup VII (Xu et al., 2006). Among those three genes, *SUB1A* helps rice plants to tolerate submergence (Fukao and Bailey-Serres, 2008a, b). In addition, it has also been shown that SNORKELs genes such as *SNORKEL 1* (*SK1*) and *SNORKEL 2* (*SK2)* can also induce tolerance in rice plants (deep-water floating rice) by inducing GA for rapid internode elongation to rescue the plants from drowning (Hattori et al., 2009). These SNORKELs also belong to ERF subgroup VII. In rice coleoptiles, a number of ERFs such as *ERF60, ERF67*, and *ERF68* genes have been shown to be upregulated under anoxic conditions and also belong to the ERF subgroup VII (Lin et al., 2019). These data favor the understanding that rice coleoptile elongation is promoted by ethylene during submerged conditions. Besides, it has been shown that auxin also plays a key role in coleoptile elongation during submergence (Ishizawa and Esashi, 1983; Wu and Yang, 2020). Auxin-dependent differential growth in rice coleoptile is shown to be due to the effect of the TF auxin response factor 1 (*OsARF1*) (Waller et al., 2002). Likewise, TF WRKY is also involved in the regulation of tolerance to rice under submergence (Viana et al., 2018). High accumulation of WRKY in both shoots and roots was observed in response to submergence. The expression of *OsWRKY11* and *OsWRKY56* was more than 100-fold in comparison to controls in the root tissues. These results support the importance of TFs in the regulation of tolerance of rice plants under submerged/flooding conditions. Recent studies of the roles of auxin in rice coleoptile elongation suggest that it plays a key role in the cell division and tropism of rice coleoptiles under submergence (Wu and Yang, 2020).

For such transcriptional regulation, the expression of a specific TF can regulate the expression of a number of specific sets of stress-responsive genes by binding to their cognate *cis*-acting elements in the promoters of specific genes (Mizoi et al., 2012). However, there are very limited studies performed on the *cis*-element enrichment analysis in the promoter of such stress-responsive genes. The studies so far revealed the identification of a number of putative *cis*-acting elements that can be potentially associated with TF families such as ARF, ERF, MYB, WRKY, bZIP, E2F, and ZnF (Mohanty et al., 2012; Lakshmanan et al., 2014; Sharma et al., 2018). This association of TFs also provides useful potential links with different hormonal signaling as it regulates the downstream genes through interactions with other regulatory molecules of signaling pathways (Song et al., 2005).

The genome-wide gene expression profile generated by microarray/transcriptome analyses on rice germination and coleoptile elongation under hypoxia/anoxia have revealed the involvement of a common molecular mechanism that is associated with carbohydrate metabolism, fermentation, hormone induction, cell division, and expansion (Lasanthi-Kudahettige et al., 2007; Shingaki-Wells et al., 2011; Narsai et al., 2015; Hsu and Tung, 2017). However, the level of tolerance to submergence/flooding during germination and coleoptile elongation could be due to the natural variation. The molecular basis of this variation due to transcriptional regulation is unknown. Hence, the aim was to analyze and identify the specific promoter architecture of gene expression data of different groups of diverse rice genotypes that display variation in the rate of coleoptile elongation under submerged conditions (Hsu and Tung, 2017). These analysis can provide a hypothesis on the key TFs that are associated with genetic variation of different genotypes of rice germination and coleoptile elongation. Further, it can be linked to common and/or unique transcriptional modules associated with different genotypes.

## Materials and Methods

### Extraction of Promoter Sequences of Common Genes Upregulated in Different Groups of Diverse Genotypes of Rice Tolerant to Submergence

Common upregulated genes in the coleoptiles of different genotype groups of rice under submerged conditions were identified from published RNA-sequencing data (Hsu and Tung, 2017). The five diverse rice genotypes-the *japonica* variety Nipponbare, two recombinant inbred lines F291 and F274-2a generated from a cross between Nipponbare and IR64, and two natural genotypes originated from Southeast Asia [8391 from Laos (IRGC 94599) and 8753 from Indonesia (IRGC 54313)]-were used for the promoter architecture analysis. To identify the promoter *cis*-element content of the common upregulated genes of different rice genotypes based on their extent of tolerance in terms of elongation of coleoptiles, common genes upregulated in different groups of genotypes (grouping done by Hsu and Tung, 2017) were extracted. The analysis was performed for all five tolerant genotypes (Nipponbare, F291, F274-2a, 8391, and 8753), which have 23 upregulated genes (Suppplementary Table 1); four highly tolerant genotypes (F291, F274-2a, 8391, and 8753), which have 16 upregulated genes (Suppplementary Table 2); and two extremely tolerant natural genotypes (8391 and 8753), which have 27 upregulated genes (Suppplementary Table 3). The promoter sequences [-1000, +200 nucleotide] relative to the experimentally verified Transcription Start Site (TSS) were extracted for these common upregulated genes from our in-house rice promoter sequence database (Mohanty et al., 2012).

To validate the identification of promoter architecture analysis of the three groups of genotypes that have varying degrees of tolerance to submergence, two additional analyses were performed. In one of the analysis that belongs to the intermediate tolerant group, two highly tolerant genotypes (F291 and F274-2a) that have 24 upregulated genes (Suppplementary Table 4) were analyzed to compare with the group that has four highly tolerant genotypes (F291, F274-2a, X8391, and X8753). In the other analysis, two genotypes (Nipponbare and F274-2a) that have 84 upregulated genes (Suppplementary Table 5) were analyzed for comparison with the group that has five tolerant genotypes (Nipponbare, F291, F274-2a, X8391, and X8753) from different backgrounds. The comparisons of the two groups of genotypes with respective similar groups of genotypes will support the analysis.

### Identification of Putative *cis*-Elements and Associated TFs

**FIGURE 1:**
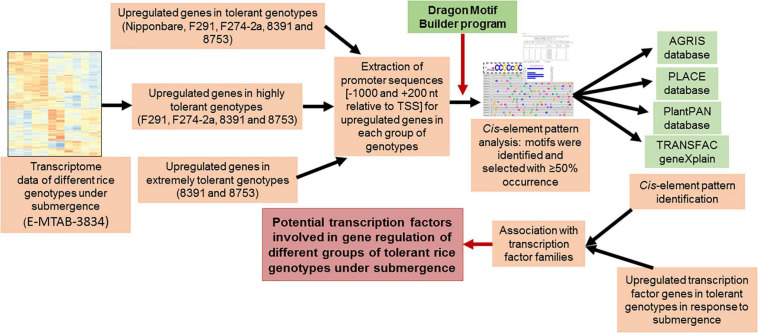
Summary of the methods used for the identification of the potential transcription factors in different groups of diverse rice genotypes tolerant to submergence. Promoter sequences of upregulated genes in different groups of genotypes that have different degrees of tolerance to submergence were extracted from our in-house rice promoter database, followed by identification and analysis of putative *cis*-elements by examining their presence in different TF binding databases. Potential TFs were identified by linking putative *cis*-elements with their associated transcription factors.

Putative *cis*-elements were identified in the promoter regions of each set of common upregulated genes by the “The Dragon Motif Builder program” having EM2 option with a threshold value of 0.875 (Huang et al., 2005), which is similar to our earlier detection (Mohanty et al., 2012, 2016; Lakshmanan et al., 2014). The program identified a total of 30 motifs having a length of 8–10 nucleotides for each set of common genes. Motifs were selected having a threshold value of 10^–3^ and more than 50% occurrence. The biological significance of these motifs was verified by Transcription Factor Binding databases such as TRANSFAC (Matys et al., 2003^1^), PLACE database (Higo et al., 1999^2^), AGRIS (Davuluri et al., 2003; Yilmaz et al., 2011^3^), and PlantPAN 3.0 (Chow et al., 2019^4^). Putative *cis*-elements in the promoters of the upregulated genes were identified based on their sites for different plant TFs present in plant genes with a minimum sequence length of five nucleotides. The cutoffs for core and matrix similarities were more than 75%. TF genes, significantly upregulated, were identified from the RNA-sequencing data (Hsu and Tung, 2017) and annotated based on the RAP genome annotations (Itoh et al., 2007; Tanaka et al., 2008^5^). The methods used to analyze the data have been summarized and presented in Figure 1.

## Results

### Identification of Putative *cis*-Elements in the Upregulated Genes of Tolerant, Highly Tolerant, and Extremely Tolerant Diverse Rice Genotypes in Response to Submergence

Promoter regions of common genes upregulated in diverse genotypes of rice germination and coleoptile elongation in response to submergence tolerance contain *cis*-elements/TF binding sites that are responsible for the regulation of genes associated with various hormone signaling as well as metabolic pathways. Our analysis identified a number of common putative *cis*-elements in the promoter regions of the upregulated genes in tolerant (Nipponbare, F291, F274-2a, 8391, and 8753), highly tolerant (F291, F274-2a, 8391, and 8753), and extremely tolerant (8391 and 8753) genotypes. Putative *cis*-elements that are present in all genotypes are found to be associated with many known TFs such as MYB, bZIP, AP2/ERF, ARF, WRKY, ZnF, MADS-box, NAC, AS2, DOF, E2F, ARR-B, and HSF (Tables 1-3). Interestingly, both highly tolerant and extremely tolerant genotypes, in addition to the above binding sites for the associated TFs, also possess unique binding sites that are associated with TFs such as HY5 (bZIP), GBF3, 4, and 5 (bZIP), DPBF-3 (bZIP), ABF2, ABI5, bHLH (basic helix-loop-helix), and BES/BZR (BR-responsive TFs) (Table 2). Moreover, the extremely tolerant genotypes that have highly elongated coleoptiles comprise more specific binding sites that are associated with bHLH TFs such as BEE2, BIM1, BIM3, BM8, and BAM8 and bZIP TF ABF1 (Table 3). Hence, the following TF regulatory modules are identified as transcriptional activators/repressors that could be involved in controlling the regulation of germination and variation in coleoptile elongation of diverse rice genotypes in response to submergence tolerance.

**TABLE 1:** Potential putative *cis*-elements identified in the promoters of upregulated genes in tolerant genotypes (Nipponbare, F291, F274-2a, 8391, and 8753).

***Cis*-elements**	**Motifs**	**Associated TFs**	% (TIC), *E*-value*
AT-hook/PE1-like	TTTTTTTTA	MYB (PF1)	61 (16.56), 1e-004
GT-element-like	CTGAAAAAG	MYB (GT-1/GT-3b)	65 (12.79), 3e-004
GARE-like	CAACAACA	MYB (R1, R2R3)	74 (11.70), 0e+000
MYB-box-like	GGTGGGCG	MYB (R2R3)	78 (10.89), 3e-004
	CAACAACA	MYB (R2R3)	74 (11.70), 0e+000
	GACAAATT	MYB (R2R3)	65 (12.58), 7e-004
	CAAAACCA	MYB (R2R3)	61 (13.34), 4e-004
As-1/ocs-like	CGTTGATC	bZIP (Gr. D, I, S)	70 (11.38), 1e-004
	CTGAAAAAA	bZIP (Gr. D, S)	65 (12.79), 3e-004
GCN4 motif	CAACAACA	bZIP (RISBZ1, Gr. G)	74 (11.70), 0e+000
	GACAAATT	bZIP (RISBZ1, Gr. G)	65 (12.58), 6e-004
CAMTA5 binding site-like	CCACACAA	bZIP (CAMTA5)	65 (12.57), 6e-004
GCC-box-like	AGTGGGCG	ERF (I, IV, VII, X)	78 (10.89), 3e-004
	CGCCGCCG	ERF (I, IV, VII, X)	70 (14.42), 9e-005
	CGCCGCCTC	ERF (I, IV, VII, X)	57 (14.27), 2e-004
ERE-like	GCCGAGAG GCGGCCATT TGCTCGCCG	ERF/RAP2.3, RAP2.10 (Gr. III) ERF/RAP2.2, RAP2.3 (Gr. III) ERF/RAP2.6, RAP2.10 (Gr. VII) ERF/RAP2.6 (Gr. VII)	65 (11.42), 3e-004 52 (12.47), 4e-004 52 (13.56), 2e-004
RAV1a-like	CAACAACA	ERF/RAV1 (Gr. II, III and RAV1)	74 (11.70), 0e+000
CRT/DRE-like	CGCCGCCTC	ERF (Gr., III, IV)	57 (14.27), 2e-004
JA response element-like	CTTTGATC	ERF (ERF Gr. VI, VIII, IX)	70 (11.38), 1e-004
ABRE-like	ATTTAGCG TGCTTGCCG	ABI3 (B3 domain of AP2/) ABI3 (B3 domain)	61 (12.23), 2e-004 52 (13.66), 2e-004
ABR-binding site-like	GCCGAGAG TGCTCGCCG	AP2-like ABA repressor 1 (ABR1) AP2-like ABA repressor 1 (ABR1)	65 (11.42), 3e-004 52 (13.66), 2e-004
Ethylene-insensitive 3 binding site-like	AAATGCAAA GAATGCAAA	EIL3 (EIN3) EIL3 (EIN3)	61 (13.85), 9e-005 61 (13.85), 9e-005
Aux-RE-like	CAACAACA ACGAGACC	ARF1 ARF1, ARF5, ARF7	74 (11.70), 0e+000 52 (11.65), 3e-004
W-box-like	GTCAAATT	WRKY (Gr. I, IIa, IIc, III)	56 (15.03), 9e-005
Zing finger binding site-like	CAACAACA GCCGAGAG	ZnF (C2H2-type) ZnF (C2H2-type)	74 (11.70), 0e+000 65 (11.42), 3e-004
IDD binding site-like	CAACAACA CCACACAA	IDD 2, 4, 5, 7 (ZnF) IDD (ZnF)	74 (11.70), 0e+000 65 (12.57), 6e-004
Zinc-finger-binding site-like	AGTGGGCG	STZ (Salt tolerant zinc finger)	78 (10.89), 3e-004
DRE-like	CAACAACA GCCGAGAG AAATGCAAA TGCTTGCCG TGCTCGCCG ACGAGACC ACGAGACC	NAC (38) NAC (65) NAC (11, 45) NAC (58) NAC (62) NAC (NAM) NAP,(NAC-like, activated by AP_3_/P_1)_	74 (11.70), 0e+000 65 (11.42), 3e-004 61 (13.85), 9e-005 52 (13.66), 2e-004 52 (13.66), 2e-004 52 (11.65), 3e-004 52 (11.65), 3e-004
AAAAG/CTTTT-element-like	CTGAAAAAA CTGAAAAAG	DOF (DOF1/4/11/22) DOF (DOF1/4/11/22)	65(12.79), 3e-004 65(12.79), 3e-004
Heat shock binding factor element-like	GCCGAGAG ATTTAGCG TTCTTCCTG	HSF(HSF3) HSF(HSFA4A, HSF7) HSF (HSFA6B HSFB4, HSF7)	65 (11.42), 3e-004 61 (12.23), 2e-004 52 (13.96), 3e-004
S2-binding site-like	CCACACAA GACAAATT CTGAAAAAG ATTTAGCG GCGGCCATT TTCTTCCTG	AS2 (LBD16, 19) AS2 (LBD16) AS2 (LBD16, 18) AS2 (LBD2) AS2 (LBD23) AS2 (LBD30)	65 (12.57), 6e-004 65 (12.58), 7e-004 65 (12.79), 3e-004 61 (12.23), 2e-004 52 (12.47), 4e-004 52 (13.96), 2e-004
E2F binding site-like	ATTTAGCG ATGGGACT GCGGCCATT	E2F-like protein, E2Fa, E2Fc (DEL1) E2F (DEL1) E2F (DEL2)	61 (12.23), 2e-004 52 (12.87), 3e-004 52 (12.47), 4e-004
TCP binding site-like	ACGAGACC	TCP (TCP 3, 24)	52 (11.65), 3e-004
HD-ZIP binding site-like	AAATGCAAA	HD-ZIP (ATHB4)	61 (13.85), 9e-005
ARR-14 binding element-like	ACGAGACC	ARR-B (ARR14)	52 (11.65), 3e-004
CArG box-binding site-like	GACAAATT GCCGAGAG AAATGCAAA ATTTAGCG	MADS-box (AGL 6, 15) MADS-box (AGL 95) MADS-box (AGL16) MADS-box (AGL16)	65 (12.58), 6e-004 65 (11.42), 3e-004 61 (13.85), 9e-005 61 (12.23), 2e-004
GATA binding site-like	ACGAGACC GCGGCCATT	GATA1 GATA1	52 (11.65), 3e-004 52 (12.47), 4e-004
DBP-binding site-like	TAAATATA	DBP1	56 (13.75), 1e-005
TATA-box-like	TAAATATA	TBP	56 (13.75), 1e-005

% = percent occurrence among all upregulated genes, TIC = total information content of homology, *E*-value* = *E*-value of homology with promoter database entry.

**TABLE 2:** Potential putative *cis*-elements identified in the promoters of upregulated genes in highly tolerant genotypes (F291, F274-2a, 8391, and 8753).

***Cis*-elements**	**Motifs**	**Associated TFs**	%(TIC), *E*-value*
AT-hook/PE1-like	TTAAAAAC TTAAAAAATA TATTAAAAAA TTAATTTTT	MYB (PF1) MYB (PF1) MYB (PF1) MYB (PF1)	63 (13.49), 2e-004 63 (16.52), 2e-005 63 (15.86), 5e-005 56 (15.46), 1e-004
GT-element-like	CAACCACA ATTTGATTT CCAACCAA TTTGTATTTA TGCATGTA	MYB (GT-1) MYB (GT-1) MYB (GT-1) MYB (GT-3b) MYB (GT-3a)	81 (11.63), 3e-004 81 (13.98), 5e-005 69 (11.71), 1e-004 63 (13.49), 2e-004 50 (13.95), 3e-005
GARE-like	CCAACCAA	MYB (R1, R2R3)	69 (11.71), 1e-004
MYB-box-like	TAAAACAAA TAAAACAGA AAACCACA CAACCACA GCTAGCTAGA CAAGCTGC CCAAACAA CCAACCAA	MYB (R2R3) MYB (R2R3) MYB (R2R3) MYB (R2R3) MYB (R2R3) MYB (R2R3) MYB (R2R3) MYB (R2R3)	94 (12.79), 1e-004 94 (12.79), 1e-004 81 (11.63), 3e-004 81 (11.63), 3e-004 81 (12.81), 3e-004 69 (12.41), 1e-004 69 (11.71), 1e-004 69 (11.71), 1e-004
MYB-box related-like	AAGCTGAG TTAATTTTT	MYB-like (CDC5) MYB-like (CCA1, CDA-1)	69 (11.15), 9e-005 56 (15.46), 1e-004
As-1/ocs-like	ATTTGATTT TGAAGCTT GATCGTGA	bZIP (Gr. D, I, S) bZIP (Gr. D, I, S) bZIP (Gr. D, I, S)	81 (13.98), 5e-005 69 (12.23), 6e-005 56 (13.61), 5e-005
ABRE-like	CAAGCTGC GATCGTGA TGCATGTA	bZIP (Gr. A) bZIP (Gr. A) bZIP (Gr. A)	69 (12.41), 1e-004 56 (13.61), 5e-005 50 (13.95), 3e-005
GCN4 motif-like	TAAAACAAA CCAAACAA AGAAAGTG	bZIP (RISBZ1, Gr. G) bZIP (RISBZ1, Gr. G) bZIP (RISBZ1, Gr. G)	94 (12.79), 1e-004 69 (11.71), 1e-004 50 (12.42), 5e-004
HY-5 binding site-like	ATTTGATTT	bZIP (Gr. H)(HY5)	81 (13.98), 5e-005
G-box-like	GATCGTGA TGCATGTA	bZIP (Gr. G) (GBF3, 5, 6) bZIP (Gr. G) (GBF3, 5)	56 (13.61), 5e-005 50 (13.95), 3e-005
CAMTA5 binding site-like	GATCGTGA	bZIP (CAMTA5)	56 (13.61), 5e-005
ABRE-like (DPBF binding site-like)	GATCGTGA TGCATGTA	bZIP (DPBF-3) bZIP (DPBF-3) (Opaque-2)	56 (13.61), 5e-005 50 (13.95), 3e-005
ABRE-like	GATCGTGA	ABI5 (bZIP)	56 (13.61), 5e-005
ABRE-like	GATCGTGA	ABF2 (bZIP)	56 (13.61), 5e-005
ERE-like	GCTCCATC	ERF/RAP 2.4 (Gr. III)	69 (13.21), 8e-005
JA response element-like	ATTTGATTT AAGTCAAA	ERF (Gr. VI, VIII, IX) ERF (Gr. VI, VIII, IX)	81 (13.98), 5e-005 69 (13.75), 4e-004
ABRE-like	CAAGCTGC GCATGGGC TGCATGTA	ABI3/V1P1 (B3 domain of AP2/ERF) FUS3, (Similar to VP1/ABI3-like proteins) FUS3, (Similar to VP1/ABI3-like proteins)	69 (12.41), 1e-004 69 (11.61), 5e-005 50 (13.95), 3e-005
B3-binding site-like	AGAAAGTG	AP2/B3-like	50 (12.42), 5e-004
Ethylene-insensitive 3 binding site-like	TCTTCCAT	EIL3 (EIN3)	50 (13.56), 6e-005
Aux-RE-like	TAAAACAAA CCAAACAA TTTGTATTTA	ARF1 ARF1 ARF1	94 (12.79), 1e-004 69 (11.71), 1e-004 63 (13.49), 2e-004
W-box-like	ATTTGATTT AAGTCAAA	WRKY (Gr. I, IIa, IIc, III) WRKY (Gr. I, IIa, IIc, III)	81 (13.98), 5e-005 69 (13.75), 4e-004
Zinc-finger-binding site-like	ATTTGATTT GATCGTGA	ZnF (ZCT1, ZCT2, ZCT3) ZnF (GAL3)	81 (13.98), 5e-005 56 (13.61), 5e-005
Zing finger binding site-like	TAAAACAAA CCAAACAA	ZnF (C2H2-type) ZnF (C2H2-type)	94 (12.79), 1e-004 69 (11.71), 1e-004
IDD binding site-like	TAAAACAAA CCAAACAA	IDD2, 4, 5, 7 (ZnF) IDD2, 4, 5, 7 (ZnF)	94 (12.79), 1e-004 69 (11.71), 1e-004
DRE-like	CAAGCTGC TGAAGCTT TCATGGGC AAGCTGAG GATCGTGA AGAAAGTG TGCATGTA	NAC (5, 45, 62, 71, 96) VND (2, 3, 4, 6) SND3 NAC (45, 58)VND 6 NAC (57, 58, 87, 92) CUC3 NAC (45) NAC (46, 47, 55, 58, 92) NAP(NAC-like, activated by AP_3_/P_1_) NST1 (NAC) ATAF1NAC (5, 45, 62, 7, 96) VND (2, 3, 4, 6) NAC (46, 57, 78) CUC1, CUC2	69 (12.41), 1e-004 69 (12.23), 6e-005 69 (11.61), 5e-005 69 (11.15), 9e-005 56 (13.51), 5e-005 50 (12.42), 5e-004 50 (13.95), 3e-005
AAAAG/CTTTT-element-like	TATTAAAAGA AGAAAGTG	DOF (DOF1/4/11/22) OBP3 DOF-type zinc finger	63 (15.86), 5e-005 50 (12.42), 5e-004
Heat shock binding factor element-like	AGAAAGTG	HSF (HSFB2A)	50 (12.42), 5e-004
S2-binding site-like	AAACCACA GCTCCATC GATCGTGA	AS2 (LBD16, 19) AS2 (LBD13) AS2 (LBD2, 19)	81 (11.63), 3e-004 69 (13.21), 8e-005 56 (13.61), 5e-005
TCP binding site-like	CAACCACA GCTCCATC	TCP (TCP 15, 16) TCP (TCP3)	81 (11.63), 3e-004 69 (13.21), 8e-005
HD-ZIP binding site-like	CAACCACA ATTTGATTT TGCATGTA	HD-ZIP (ATHB7) HD-ZIP (ATHB6, 7, 12 and 13) HD-ZIP (ATHB4)	81 (11.63), 3e-004 81 (13.98), 5e-005 50 (13.95), 3e-005
ARR10-binding element-like	ATTTGATTT	ARR-B (ARR10)	81 (13.98), 5e-005
CArG box-binding site-like	TAAAACAAA ATTTAGCG TCTTCCAT AGAAAGTG	MADS (AGL 6, 15) MADS (AGL 6, 15) MADS (AGL 16) MADS box (AGL 6, 15, and 16)	94 (12.79), 1e-004 65 (12.58), 6e-004 50(13.56), 6e-005 50 (12.42), 5e-004
GATA binding site-like	CAACCACA CCAACCAA	GATA1 GATA1	81 (11.63), 3e-004 69 (11.71), 1e-004
E-box-like	GATCGTGA TGCATGTA	BES/BZR (BES1/BZR1) BES/BZR (BES/BZR1 homologue 2 and 3)	56 (13.61), 5e-005 50 (13.95), 3e-005
E-box-like/G-box-like	GATCGTGA TGCATGTA	bHLH (Gr. III, VII) bHLH (Gr. III, VII)	56 (13.61), 5e-005 50 (13.95), 3e-005
MBF1 binding element	TCCTCCTC	MBF1 (MBF1c)	56 (13.42), 2e-004
BPC-binding site-like	TTTCTCTC GTTCTCTC AGAAAGTG	BPC (BPC1, 6) BPC (BPC1) BPC (BPC1)	81 (12.26), 3e-004 81 (12.26), 3e-004 50 (12.42), 5e-004
DBP-binding site-like	TATTAAAAAA TTAATTTTT	DBP DBP	63 (15.86), 5e-005 56 (15.46), 1e-004
TATA-box-like	GTAATTATA	TBP	56 (15.47), 2e-005

**%** = percent occurrence among all upregulated genes, TIC = total information content of homology, *E*-value* = *E*-value of homology with promoter database entry.

**TABLE 3:** Potential putative *cis*-elements identified in the promoters of upregulated genes in extremely tolerant genotypes (8391 and 8753).

***Cis*-elements**	**Motifs**	**Associated TFs**	% (TIC), *E*-value*
AT-hook/PE1-like	AAAAATAT ACAAAAAAAA	MYB (PF1) MYB (PF1)	67 (13.84), 3e-004 52 (16,17), 0e+000
GT-element-like	CATTTGTT AGACGTGG	MYB (GT-3) MYB (GT-3a)	63 (11.57), 6e-005 63 (10.82), 9e-005
GARE-like	CATTTGTT	MYB (R1, R2R3)	63 (11.57), 6e-005
MYB-box-like	TGCTACTG AGAACATAG CATTTGTT ACAAAAAAAA	MYB (R2R3) MYB (R2R3) MYB (R2R3) MYB (R2R3)	81 (11.45), 2e-005 70 (12.40), 2e-004 63 (11.57), 6e-005 52 (16.17), 0e+000
As-1/ocs-like	AAATTTGA CATTGTGA TTGAAAAAT AGACGTGG	bZIP (Gr. D, I, S) bZIP (Gr. D, S) bZIP (Gr. D, S) bZIP (Gr. D, I, S)	67 (13.30), 4e-004 67 (12.12), 9e-005 63 (14.06), 2e-004 63 (10.82), 9e-005
C-box-like/G-box-like	AGACGTGG AGACGTGG AGTCGTGG	bZIP (Gr. A, B, H) bZIP (Gr. G) (GBF 1, 3, 5, 6) bZIP (Gr. G) (GBF 6) bZIP (Gr. G), (CPRF5, CPRF6,, CPRF7)	63 (10.82), 9e-005 63 (10.82), 9e-005 63 (10.82), 9e-005
HY-5 binding site-like	AGACGTGG	bZIP (Gr. H) (HY5)	63 (10.82), 9e-005
GCN4 motif	CATTTGTT ACAAAAAAAA	bZIP (RISBZ1, Gr. G) bZIP (RISBZ1, Gr. G)	63 (11.57), 6e-005 52 (16.17), 0e+000
CAMTA5 binding site-like	AGTCGTGG	bZIP (CAMTA5)	63 (10.82), 9e-005
ABRE-like (DPBF binding site-like)	AGACGTGG	bZIP (DPBF-3)	63 (10.82), 9e-005
ABRE-like	AGACGTGG AGTCGTGG	ABI5 (bZIP) ABI5 (bZIP)	63 (10.82), 9e-005 63 (10.82), 9e-005
ABRE-like	AGACGTGG AGTCGTGG	ABF1, ABF2 (bZIP) ABF2 (bZIP)	63 (10.82), 9e-005 63 (10.82), 9e-005
ERE-like	AGACGTGG AGTCGTGG	ERF/RAP2.3, (Gr. III) ERF/RAP2.3, RAP2.6 RAP2.10 (Gr. III), ERF (Gr. VII)	63 (10.82), 9e-005 63 (10.82), 9e-005
DRE/CRT-like	AGTCGTGG	ERF/DDF1, CBF3, CBF4, (Gr. III, IV)	63 (10.82), 9e-005
ABRE-like	AGAAAGTA	AP2/B3 (Gr. II) Related to RAV2	63 (12.66), 8e-005
JA response element-like	AAATTTGA	ERF (Gr. VI, VIII, IX)	67 (13.30), 4e-004
Ethylene-insensitive 3 binding site-like	AGAACATAG	EIL3 (EIN3)	70 (12.40), 2e-004
Aux-RE-like	CATTTGTT TTGAAAAAT	ARF1 ARF16	63 (11.57), 6e-005 63 (14.06), 2e-004
W-box-like	TTGAAAAAT	WRKY (Gr. I, IIa, IIc, III)	63 (14.06), 2e-004
Zinc finger binding element-like	TCAAATTAA	ZnF (ZCT1, ZCT2, ZCT3)	59 (13.81), 1e-005
Zing finger binding site-like	TTGAAAAAT AGAAAGTA GAAATCCT TAGTAGTA	ZnF (C2H2-type) ZnF (C2H2-type) ZnF (C2H2-type) ZnF (C2H2-type)	63 (14.06), 2e-004 63 (12.66), 8e-005 63 (11.69), 2e-004 52 (13.63), 2e-005
IDD binding site-like	TTGAAAAAT	IDD 2, 5 (ZnF)	63 (14.06), 2e-004
DRE-like	TGCTACTG CATTGTGA AGACGTGG AGTCGTGG AGACGTGG AGTCGTGG	NAC (62, 96) NAC (46) NAC (34, 42, 46, 47, 55, 70, 94) NAC (45, 46, 47) NAC (34, 42, 45, 55, 58) NAC (NAM) NAP (NAC-like, activated by AP_3_/P_1)_ NAP (NAC-like, activated by AP_3_/P_1)_	81 (11.45), 2e-005 67 (12.12), 9e-005 63 (10.82), 9e-005 63 (10.82), 9e-005 63 (10.82), 9e-005 63 (10.82), 9e-005
ATAF1-binding site-like	AGTCGTGG	ATAF1(NAC)	63 (10.82), 9e-005
AAAAG/CTTTT-element-like	AGAAAGTA	DOF (DOF 4.5)	63 (12.66), 8e-005
Heat shock binding factor element-like	AGAACATAG CATTGTGA GAAATCCT	HSF (HSFB2A HSF) HSF (HSFB2A) (HSFA8)	70 (12.40), 2e-004 67 (12.12), 9e-005 63 (11.69), 2e-004
S2-binding site-like	CATTGTGA AGACGTGG AGTCGTGG ACAAAAAAAA	AS2 (LBD16, 18, 19) AS2 (LBD2) AS2 (LBD23) AS2 (LBD16)	67 (12.12), 9e-005 63 (10.82), 9e-005 63 (10.82), 9e-005 52 (16.17), 0e+000
HD-ZIP binding site-like	AAAATTAG	ATHB3	55 (12.95), 5e-005
ARR14-binding element-like	AGAACATAG	ARR-B (ARR14)	70 (12.40), 2e-004
CArG box-binding site-like	ACAAAAAAAA AGAAAGTA AAGATTGCA	MADS (AGL 15, 16) MADS (AGL 6) MADS (AGL 6)	52 (16.17), 0e+000 63 (12.66), 8e-005 74 (12.50), 2e-004
E-box-like	AGACGTGG	BES/BZR (BES1/BZR1)	63 (10.82), 9e-005
E-box-like/G-box-like	AGACGTGG AGTCGTGG	bHLH (Gr. III, VII) bHLH (Gr. III, VII)	63 (10.82), 9e-005 63 (10.82), 9e-005
E-box-like	AGACGTGG	BEE2 (bHLH) BIM1, BIM3 (bHLH)	63 (10.82), 9e-005
BBRE-element-like/G-box-like	AGACGTGG	BAM8 (bHLH): BAMs	63 (10.82), 9e-005
BRRE-element/G-box-like	AGACGTGG	BEH4 (bHLH)	63 (10.82), 9e-005
DBP-binding site-like	TCAAATTAA	DBP	59 (13.81), 1e-005
TATA-box-like	TAAATATA	TBP	56 (13.75), 1e-005

% = percent occurrence among all upregulated genes, TIC = total information content of homology, *E*-value* = *E*-value of homology with promoter database entry.

#### MYB Regulatory Module

The promoter analysis of common upregulated genes in the tolerant, highly tolerant, and extremely tolerant genotypes identified high enrichment of a number of MYB-associated putative *cis*-elements such as AT-hook/PE1-like, GT-element-like, gibberellic acid response element (GARE)-like, and MYB-box-like. The MYB-box-like elements were highly enriched in the highly tolerant genotypes and, besides the promoters, also possess MYB-box related-like elements associated with MYB-like (CDC5) and MYB-like (CCA1, CDA-1) TFs (Tables 1-3). These elements are most likely associated with the upregulation of a number of MYB genes (Table 4) such as *Os12g0125000* (MYB-like DNA-binding domain-containing protein), *Os11g0128500* (MYB-like DNA-binding domain-containing protein), *Os05g0553400* (Similar to MYB-related TF), *Os01g0298400* (Putative typical P-type R2R3 MYB protein), *Os12g0567300* (MYB TF domain-containing protein, R2R3 MYB), *Os01g0874300* (Putative MYB-related protein, MYB2), and *Os05g0140100* (R2R3 MYB TF) (Table 4).

**TABLE 4:** List of upregulated transcription factors with potential significance to the identified putative *cis*-elements among upregulated genes in five genotypes from diverse background in response to submergence tolerance.

**TF Family**	Locus_ID (Annotation)*	Fold increase**
MYB		
	Os12g0125000 (MYB-like DNA-binding domain-containing protein)	5.90
	Os11g0128500 (MYB-like DNA-binding domain-containing protein)	5.57
	Os05g0553400 (Putative MYB-related transcription factor)	5.14
	Os01g0298400 (Putative typical P-type R2R3 MYB protein)	4.55
	Os12g0567300 (MYB transcription factor domain-containing protein: R2R3 MYB)	3.24
	Os01g0874300 (Putative MYB-related protein; Putative MYB2)	3.20
	Os05g0140100 (R2R3 MYB transcription factor)	2.44
bZIP		
	Os11g0152700 (BZIP transcription factor: transcription factor HBP-1)	5.48
	Os12g0547600 (Calmodulin-binding protein, putative, expressed)	3.10
	Os05g0129300 (bZIP transcription factor)	2.51
ERF		
	Os01g0968800 (DREB transcription factor like)	Infinite
	Os06g0127100 (Dehydration-responsive element-binding protein 1C)	Infinite
	Os01g0868000 (AP2/ERF transcription factor like)	6.86
	Os09g0522100 (Similar to Dehydration-responsive element-binding protein 1H)	6.44
	Os01g0140700 (Similar to RAV family protein: AP2/ERF and B3 domain-containing protein)	6.16
	Os02g0677300 (Similar to CRT/DRE binding factor 1)	4.71
	Os02g0654700 (AP2/ERF family protein)	4.65
	Os02g0656600 (Similar to DRE binding factor 2B)	4.52
	Os05g0549800 (Similar to DNA-binding protein RAV1)	4.17
	Os03g0184500 (B3 domain-containing protein: ABIVP1 transcription factor)	3.58
	Os01g0693400 (RAV family protein)	2.89
ARF		
	Os12g0601400 (Auxin-responsive protein IAA31)	2.51
WRKY		
	Os05g0583000 (Similar to WRKY transcription factor 8)	Infinite
	Os05g0537100 (WRKY transcription factor 10, WRKY transcription factor 7)	4.27
	Os01g0750100 (Similar to WRKY transcription factor 13)	2.71
ZnF		
	Os03g0437200 (C2H2-type zinc finger protein, abscisic acid-induced antioxidant defense, Water stress and oxidative stress tolerance)	6.82
	Os03g0820300 (C2H2 transcription factor protein)	6.16
	Os12g0113700 (Zinc finger, C3HC4 type family protein)	6.04
	Os02g0646200 (Zinc finger, B-box domain-containing protein)	4.43
	Os10g0456800 (CHY zinc finger family protein)	2.81
	Os01g0303600 (Zinc finger, RING/FYVE/PHD-type domain-containing protein)	3.82
	Os06g0340200 (Zinc finger, RING-CH-type domain-containing protein)	3.33
	Os03g0329200 (Zinc finger CCCH domain-containing protein 23)	3.27
	Os03g0764100 (Zinc finger transcription factor ZF1)	2.60
	Os09g0486500 (Zinc finger A20 and AN1 domain-containing stress-associated protein 1)	2.49
	Os05g0128200 (Zinc finger CCCH domain-containing protein 33)	2.21
NAC		
	Os11g0154500 (No apical meristem (NAM) protein domain-containing protein; NAC-domain-containing protein 90)	5.83
	Os03g0815100 (Similar to OsNAC6 protein)	4.94
	Os01g0884300 (NAC domain-containing protein 6)	3.50
	Os07g0684800 (Similar to NAM/CUC2-like protein)	3.33
	Os07g0225300 (OsNAC3 protein; NAC domain-containing protein 67)	3.27
MADS-box		
	Os04g0580700MADS-box transcription factor 17	6.07
HSF		
	Os08g0471000 (Heat stress transcription factor B-4a, HSF20)	Infinite
	Os09g0526600 (Heat stress transcription factor B-2c, HSF 3)	4.51
	Os09g0456800 (Heat stress transcription factor B-1)	4.05
	Os02g0232000 (Similar to Heat shock transcription factor 29, HSF 5)	3.67
PhD-finger		
	Os03g0302200 (PHD-finger family protein)	2.65
JAZ		
	Os10g0391400 (Jasmonate ZIM-domain (JAZ) protein, Negative regulation of JA signal transduction pathway)	7.81
	Os03g0180800 (Jasmonate ZIM-domain protein 3)	6.04
	Os08g0428400 protein (ZIM transcription factor; Jasmonate ZIM-domain protein 9)	2.49
HD ZIP		
	Os05g0129700 (Homeobox protein knotted-1-like 10)	4.82
	Os06g0140400 (Homeobox-leucine zipper protein HOX28)	4.50
	Os03g0198600 (Homeodomain-leucine zipper transcription factor)	4.46
	Os06g0140700 (Homeobox-leucine zipper protein HOX2)	3.49
	Os03g0188900 (Homeobox-leucine zipper protein HOX13)	3.21
	Os09g0528200 (Similar to Homeobox-leucine zipper protein HOX6Homeobox-leucine zipper protein HOX6)	2.83
bHLH		
	Os01g0773800 (Basic helix-loop-helix protein 185)	Infinite
	Os03g0188400 (Basic helix-loop-helix protein)	3.84
	Os07g0628500 (Basic helix-loop-helix dimerization region bHLH domain-containing protein)	3.31
	Os03g0135700 Basic helix-loop-helix transcription factor	3.16
	Os07g0143200 (Phytochrome-interacting bHLH factor)	2.44
G-box binding protein	G-box binding protein; G-box binding protein-like (B12D-like protein); Os06g0246000 protein	2.70

*Information based on RAP-DB (http://rapdb.dna.affrc.go.jp/); **based on RNA-seq data of Hsu and Tung (2017).

It has been highlighted that MYB TFs represent a major protein in rice and are involved in the transcriptional regulation of many developmental processes as well as abiotic and biotic stress conditions (Katiyar et al., 2012). Additionally, it has already been shown both experimentally and *in silico* analysis that MYB TFs that bind to MYB-box/GT-element-like elements play a key role in the transcriptional regulation of rice during germination under submergence and anoxia (Dolferus et al., 2003; Mohanty et al., 2012; Lakshmanan et al., 2014). In this analysis, MYB-box-like elements are more highly enriched compared to GARE-like elements. Under flooding/submergence/anoxic conditions, the plant hormone gibberellic acid (GA) plays a major role in rice and other plants such as barley (Gubler et al., 2002; Kaneko et al., 2002). It activates the endosperm reserve to aleurone layers for the induction of enzyme α-amylases for the hydrolysis of starch, protein, and cell wall reserve (Woodger et al., 2003; Lee et al., 2014). Under submerged/flooding conditions, *α*−amylases play a major role in providing sugar substrates by hydrolyzing starch for glycolysis and alcohol fermentation to generate ATP. Recently, Abdulmajid et al. (2020) have screened favorable rice genotypes for coleoptile elongation length sensitivity to exogenous gibberellin under submerged conditions. However, in this analysis, pyrimidine-box-like elements and GARE-like elements are less enriched compared to MYB-box-like elements, which are associated with MYB (R1, R2R3). A similar pattern was also identified in our previous analysis for the transcriptional regulation of coleoptile germination and elongation of the *japonica* rice under anoxia (Mohanty et al., 2012). It appears that submergence tolerance for rice germination may not be completely mediated by GA. Recently, it has been shown that two MYB TFs that bind to the same *cis*-element regulate the on/off switch of *α-Amy* expression (Chen et al., 2019). MYBS1 activates the expression of *α-Amy* during sugar starvation and promotes nuclear import of MYBS1, whereas MYBS2 behaves in the opposite manner during sugar provision. They have also shown that there is no enhancement of submergence tolerance in rice seedlings when the expression of MYBS2 was reduced. However, *α-Amy* is necessary for growth of rice seedlings under submergence as reduced seedling growth was observed in MYBS2-Ox lines under submergence. High enrichment of MYB-box-like element associated with R2R3 MYB and upregulation of R2R3 MYB genes in the tolerant genotypes suggest that abscisic acid (ABA) could also be playing a role together with other hormones in the germination and coleoptile elongation of rice under submergence.

#### bZIP Regulatory Module

As-1/ocs-like, GCN4 motif, and CAMTA5 binding site-like were highly enriched in the upregulated genes of tolerant, highly tolerant, and extremely tolerant genotypes (Tables 1-3). However, upregulated genes of highly tolerant and extremely tolerant genotypes possess specific ABRE-like elements associated with bZIP (Gr. A), bZIP (Gr. G) (GBF 3, 5, and 6), bZIP (DPBF-3), ABI5 (bZIP), and ABF2 (bZIP) and C-box-like/G-box-like and HY-5 binding site-like elements associated with GBF and HY5, respectively (Tables 2, 3). These binding sites could be associated with the upregulation of genes such as *Os12g0547600* (Calmodulin-binding protein, putative, expressed), *Os11g0152700* (bZIP transcription factor 79; transcription factor HBP-1), and *Os05g0129300* (bZIP transcription factor) (Table 4). bZIP TFs play a key role in plant growth, development, and abiotic and biotic stress conditions in *Arabidopsis* and rice (Zhang et al., 2015; Yang et al., 2019). Although the role of bZIP in response to abiotic stresses has been well studied in *Arabidopsis*, a few cases have been characterized in rice (Tang et al., 2012, 2016; Zhang et al., 2017; Yang et al., 2019). Moreover, substantial enrichment of bZIP-associated elements suggest the significance of ABRE-like/CAMTA5/C-box/G-box-like elements in response to ABA signaling in response to submergence. The role of bZIP in response to submergence/flooding stress in rice is not well studied yet. In rice, *OsbZIP45*, an ortholog of maize GBF1, was shown to be induced by hypoxia (de Vetten and Ferl, 1995). Later, *OsABF1*, an ABA-responsive element (ABRE) binding bZIP TF, has been shown to be induced during different abiotic stresses, such as anoxia, drought, cold, salinity, and ABA in rice seedlings (Hossain et al., 2010). ABA plays a key role in plant physiology, development, seed maturation, dormancy, and responses to a number of abiotic stress conditions such as drought, cold, and salt (Dar et al., 2017). Promoter regions of many genes possess ABRE-responsive elements that are responsible for ABA-dependent regulation. These elements interact with various ABA-responsive TFs that regulate ABA response particularly (Kim et al., 2004). Recently, induction of bZIP in response to short- and long-term hypoxia in tomato root has been observed (Safavi-Rizi et al., 2020). In the previous analysis, we identified both high enrichment of As1/ocs-like element, ABRE-like, G-box-like, GCN4-like, and CAMTA5-like in the promoters of genes upregulated in anoxia (Mohanty et al., 2012), glycolysis and fermentation (Lakshmanan et al., 2014), and in the wild-type rice cultivar (Kinmaze) during germination and coleoptile elongation (Mohanty et al., 2016). In rice, all genotypes tolerant to submergence also showed upregulation of bZIP TF and a calmodulin-binding protein. Identification of CAMTA5 binding site-like elements associated with CAMTA5 TF proposes that it could be involved in regulating auxin transport and homeostasis (Galon et al., 2010). High enrichment of ABA-regulated bZIP TFs suggests a cross-talk between sugar and ABA-signaling during germination and coleoptile elongation under submergence. Besides, there could be some mutual enhancement between ABA and ethylene and ethylene could repress ABA activity depending on the requirement.

Interestingly, binding sites associated with bZIPs such as HY5, DPBF-3, and GBF 3, 5, and 6 were identified in both highly tolerant and extremely tolerant rice genotypes. HY5 is a key integrating factor for light and ABA pathways, and it stimulates ABA signaling pathway by binding to the promoter of ABI5 (Chen et al., 2008). It also regulates cell elongation and proliferation besides its other roles in plant growth and development (Jing et al., 2013). The presence of high enrichment of ABRE-like elements associated with different bZIP TFs especially in both highly tolerant and extremely tolerant genotypes suggests a possible temporal role of ABA signaling in response to longer coleoptile elongation.

#### ERF Regulatory Module

A number of putative *cis*-elements associated with different groups of ERF TFs were identified in the promoter regions of the upregulated genes in tolerant, highly tolerant, and extremely tolerant genotypes in response to submergence (Tables 1-3). Genes in tolerant genotypes were highly enriched with GCC-box−like elements associated with Groups VI, VIII, and IX ERF TFs; ERE-like elements associated with Groups I, IV, and VII TFs; jasmonic acid response element (JARE)-like elements associated with Gr. VI, VIII, and IX ERF TFs; and RAV-1-like element associated with ERF/RAV1 (Gr. II) TFs (Table 1). There is also enrichment of ABRE-like elements associated with Group II ABI3 AP2/B3 related to RAV TF, CRT/DRE-like elements associated with Group IV ERF TF and ABRE-binding site-like associated with ABR1 TF (Table 1). The enrichment of *cis*-elements related to the ERF group is less compared to their presence in tolerant genotypes (Tables 2, 3). GCC-box-like elements are also absent in these groups (Tables 2, 3). However, these genes possess binding sites associated with ERF (Gr. VI, VIII, and IX), ABI3 AP2/B3 related to RAV and ERF/RAP 2.4, and ERF/RAP 2.3, 2.6, and 2.10 (Tables 2, 3). The enrichment pattern of these elements could be correlated with the upregulation of a number of ERF genes such as *Os01g0968800* (DREB transcription factor-like), *Os06g0127100* (Dehydration-responsive element-binding protein 1C), *Os01g0868000* (AP2/ERF transcription factor-like), *Os09g0522100* (Dehydration-responsive element-binding protein 1H), *Os01g0140700* (Similar to RAV2: AP2/ERF and B3 domain-containing protein), *Os02g0677300* (Similar to CRT/DRE binding factor 1), *Os02g0654700* (AP2/ERF family protein, abiotic stress response), *Os02g0656600* (DRE binding factor 2B), *Os03g0184500* (B3 domain-containing protein, ABIVP1 transcription factor), and *Os01g0693400* (RAV family protein) (Table 4).

In rice, ERF VII plays a key role in flooding, hypoxia, and submergence conditions (Bailey-Serres et al., 2012; Bui et al., 2015; Gibbs et al., 2015). *SUBMERGENCE 1A* (*Sub1A*), an ERF-type TF, regulates submergence/flooding tolerance in rice. Although the *japonica* cultivar “Nipponbare” lacks this gene (Fukao et al., 2006; Xu et al., 2006), it germinates and elongates its coleoptile under anoxia and submergence conditions. Hence, this shows that there could be some other mechanism that is independent of *SUB1A* in these genotypes to tolerate anoxia and submergence (Lee et al., 2009). An earlier evidence by Ishizawa and Esashi (1988) showed that ethylene is important for the transport of sucrose from the scutellum to the coleoptile during germination and coleoptile elongation of rice seeds. Recently, it has been reported that ethylene plays an important role in the signal transduction pathway dependent on ethylene and oxygen under hypoxia/submergence conditions. In contrast, two ERFs such as *SK1* and *SK2* are involved in submergence adaptation in deep-water rice by rapidly elongating the internodes through the action of GA response (Hattori et al., 2009).

In *Arabidopsis*, members of ERF-VIIs such as ERF71/HRE2, ERF72/RAP2.3, ERF73/HRE1, ERF74/RAP2.12, and ERF75/RAP2.2 are induced under limited oxygen conditions to regulate many hypoxia-induced genes involved in fermentation, sugar metabolism, and ethylene biosynthesis. Besides, it has been shown that RAP2.12 is present in the plasma membrane in an inactive state in the presence of oxygen and then moves to the nucleus under hypoxia/submergence conditions (Kosmacz et al., 2015). This mechanism shows the fast response of the TF to oxygen shortage in order to protect plant cells. In *Arabidopsis*, a higher survival rate under low oxygen condition was observed by overexpressing RAPs (RAP2.2, RAP2.3, and RAP2.12) (Papdi et al., 2015; Yao et al., 2017). In this analysis, GCC-box-like elements associated with ERF (I, IV, VII, and X) and ERE-like elements associated with ERF (Gr. III) such as RAP2.2, RAP2.3, RAP2.6, and RAP2.10 were identified. However, identification of moderate enrichment of RAV1a-like elements can be associated with the expression of *Os05g0549800* (AP2/ERF and B3 domain-containing protein: similar to DNA-binding protein RAV1). In *Arabidopsis*, during seed germination and early seedling development, RAV1 plays a key role in ABA signaling by repressing the expression of ABI3, ABI4, and ABI5 by binding to the 5′−CAACA−3′ motif within the promoter of ABI3, ABI4, and ABI5. These results suggest that RAV1 could also be acting as a repressor of ABA signaling. In addition to ABA inhibition, ethylene also inhibits the biosynthesis of jasmonate (JA) during coleoptile elongation of etiolated rice seedlings (Xiong et al., 2017). Although the role of ERF-VIIs has been well studied in response to submergence, it is still a complex process and the regulatory mechanism is still not clearly understood yet.

The upregulated genes in the tolerant genotypes are highly enriched with ABR1 (AP2-like ABA repressor 1) binding-site-like motifs associated with ABR1 TF belonging to AP2-domain-containing protein group X in rice. TF ABR1 functions as a negative regulator of ABA response during seed germination. The expression of this TF is induced during various abiotic stresses, such as drought, cold, and salt in *Arabidopsis* (Pandey et al., 2005). In rice, the group X *OsERFs* are also closely related to the ortholog of *ABR1* in *Arabidopsis* (Mishra et al., 2013) and show significant expression during different developmental stages and response to various abiotic stresses. Interestingly, the expression of *AtERF#111*/*ABR1* in *Arabidopsis* was shoot specific and induced under submergence and hypoxia (van Veen et al., 2016; Yeung et al., 2018). Later, the regulation of *AtERF#111* expression was suggested to be related to mechanical stress during submergence as it was regulated by WRKY18, 33, and 40 (Birkenbihl et al., 2017; Bäumler et al., 2019). Significant induction of TFs WRKY18, WRKY33, and WRKY40 is reported to be induced under both submergence and anoxia as well as wounding stress (Hsu et al., 2013; Tsai et al., 2014; Wang et al., 2015). This shows that ABR1 related to wounding/pathogen response was also induced during submergence to stimulate the immune response against the threat of wounding or pathogen infection after flooding (Bäumler et al., 2019). This TF could be involved in the immune response mechanism during rice germination and coleoptile elongation in tolerant genotypes in response to submergence.

#### EIN3/EIL1 Regulatory Module

The promoter analysis of the upregulated genes in all tolerant, highly tolerant, and extremely tolerant genotypes identified the presence of putative EIN3 binding-like elements in 50–70% of the genes (Tables 1-3). This binding site can be associated with EIN3/EIL1 TF. However, there was no expression of this TF in tolerant genotypes. This TF is the master transcriptional regulator of ethylene signaling as it is required for the activation of the ethylene pathway. It regulates the transcription of ethylene-responsive genes under different environmental and spatiotemporal conditions in *Arabidopsis* (An et al., 2010; Dolgikh et al., 2019). Besides, EIN3/EIL1 controls multiple transcriptional cascades. It also targets genes such as *ERF1*, *PIF3*, and *CBF1/2/3*, which are important regulators during different abiotic stress conditions and developmental processes (Zhang et al., 2011; Shi et al., 2012; Zhong et al., 2012). It has been reported that EIN3 is involved in the regulation of a secondary transcriptional ethylene response that includes TFs such as AP2/ERFs: ERF1, ERF5, WRKY14/47, PIF3, NAC6, and RAP2.2 (Chang et al., 2013). EIN3 binding also modulates feedback regulation of the ethylene signaling pathway and integrates between different hormone-mediated pathways. These results suggest that it could be playing a key role in rice germination and coleoptile elongation mainly to activate ethylene signaling and ERF TFs and could also act as a mediator in regulating other hormone signaling and TFs.

#### ARF Regulatory Module

Aux-RE-like elements associated with ARF TFs are identified in all tolerant, highly tolerant, and extremely tolerant genotypes, and this could be associated with the expression of ARF gene such as *Os12g0601400* (Auxin-responsive protein IAA31) (Tables 1-4). The phytohormone auxin plays a key role in plant growth and development. It has been suggested that the elongation of rice coleoptile under submerged conditions could be cooperatively regulated by the endogenous concentration of ethylene and auxin (Ishizawa and Esashi, 1983). Moreover, it was shown that external IAA addition had a positive effect on the initial elongation of coleoptile (Breviario et al., 1992), whereas ethylene enhanced the later stage of elongation (Hoson et al., 1992). Auxin mainly promotes cell division. It has been shown that coleoptile in rice elongates rapidly in response to auxin treatment (Perrot-Rechenmann, 2010). *OsARF*, a rice homolog of the ARF, was found to be positively correlated with auxin-dependent differential growth in rice coleoptiles (Waller et al., 2002). Recently, Wu and Yang (2020) have revealed the involvement of auxin signaling in regulating rice coleoptile elongation as well as regulation of carbohydrate metabolism and secondary metabolism under submergence. Besides, auxin plays a key role in cell division during coleoptile elongation in the *japonica* rice under submergence (Nghi et al., 2020). Differences in auxin transport determine the length of coleoptile while availability of higher auxin level determines the final length of coleoptile under submerged conditions. They have also claimed that the long coleoptile in rice under submergence is due to an increase in auxin transport by the influx carrier AUX1. Besides, it also regulates the expression of other TFs. The presence of Aux-RE-like elements in the promoters of upregulated genes present in all groups and significant expression of ARF gene suggest a role of ARF and auxin signaling in coleoptile elongation under submergence.

#### WRKY Regulatory Module

Promoter analysis of the upregulated genes identified enrichment of W-box-like elements associated with WRKY TF in all tolerant, highly tolerant, and extremely tolerant genotypes, and those could be associated with the upregulation of WRKY genes such as *Os05g0583000* (Similar to WRKY transcription factor 8), *Os05g0537100* (WRKY transcription factor 10, WRKY transcription factor 7), and *Os01g0750100* (WRKY transcription factor 13) (Tables 1-4). Identification of W-box-like elements also coincides with the previous identification in the genes upregulated during germination and coleoptile elongation in rice (Mohanty et al., 2012; Lakshmanan et al., 2014). WRKY plays a key role in plant growth and development, secondary metabolite biosynthesis, and response to a number of abiotic and biotic stresses (Phukan et al., 2016). Increase in the expression of WRKY has also been shown by microarray data in *Arabidopsis* (Loreti et al., 2005) and rice (Lasanthi-Kudahettige et al., 2007) in response to oxygen deficiency. The role of WRKY in the regulation of submergence tolerance in rice has also been reported by Viana et al. (2018). Also, in transgenic *Arabidopsis* expressing a sunflower WRKY, *HaWRKY76* was found to be tolerant to flooding (complete submergence) by preserving carbohydrates, mainly sucrose and starch, through the repression of fermentation pathways (Raineri et al., 2015). Upregulation of WRKYs at different time points of submergence stress was also observed in maize (Campbell et al., 2016). Similar observations were also identified in *alcohol dehydrogenase 1 (ADH1)*-deficient mutant of rice where WRKY could be playing a key role in cell survival rather than elongation (Mohanty et al., 2016). Interestingly, W-box elements have been identified in the promoter of upregulated WRKY genes such as *OsWRKY11*, *OsWRKY56*, and *OsWRKY62* during submergence (Viana et al., 2018). This shows the self-regulation of WRKY in response to submergence stress. Overall, it shows that WRKY could be playing a role by self-regulating through an on–off switch depending on the condition in response to submergence.

A number of WRKY TFs are known to be ABA responsive and are involved in ABA signaling pathways. However, in *Arabidopsis*, three WRKY genes such as *AtWRKY18*, *AtWRKY40*, and *AtWRKY60* negatively regulate ABA signaling. Among them, *AtWRKY 40* binds to the promoter of ABI4 and ABI5 and represses the expression of ABA-responsive genes (Shang et al., 2010). *ABI4* itself and *WRKY 9* positively regulate *ABI4* expression in *Arabidopsis* (Chen et al., 2013), whereas ABI3/VP1 (Feng et al., 2014) and Basic Pentacysteine (BPC) negatively regulate *ABI4* (Mu et al., 2017). However, the TF *WRKY 6* induces the expression of *RAV1* by binding to the W-box motif present in its promoter and this represses the expression of *ABI3*, *ABI4*, and *ABI5* (Huang et al., 2016).

In addition to the high enrichment of putative *cis*-elements associated with WRKY TF, high enrichment of putative *cis*-elements associated with AB3/VP1 and ABI5 was also identified. Recently, the role of WRKYs in hypoxia tolerance has been demonstrated in *Arabidopsis* (Tang et al., 2021). Through genetic and molecular experiments, it has been shown that WRKYs synergistically (WRKY33 interacts with WRKY12) increase the activation of TF RAP2.2 to increase the hypoxia tolerance in *Arabidopsis* and support the role of WRKY in hypoxia tolerance. The analysis of promoter architecture suggests its self-regulation depending on its requirement to activate other TFs to enhance submergence tolerance.

#### ZnF Regulatory Module

The promoter analysis results identified a high enrichment of zinc-finger binding element-like associated with ZnF TF in all tolerant, highly tolerant, and extremely tolerant genotypes (Tables 1-3). These elements are probably associated with the upregulation of different ZnF genes such as *Os03g0437200* (C2H2-type zinc finger protein, ABA-induced antioxidant defense, water stress, and oxidative stress tolerance), *Os03g0820300* (C2H2 transcription factor), *Os12g0113700* (Zinc finger, C3HC4-type family protein), *Os02g0646200* (Zinc finger, B-box domain-containing protein), *Os10g0456800* (CHY zinc finger family protein), *Os01g0303600* (Zinc finger, RING/FYVE/PHD-type domain-containing protein), *Os06g0340200* (Zinc finger, RING-CH-type domain-containing protein), *Os03g0329200* (Zinc finger CCCH domain-containing protein 23), *Os03g0764100* (Zinc finger transcription factor ZF1), *Os09g0486500* (Zinc finger A20 and AN1 domain-containing stress-associated protein 1), and *Os05g0128200* (Zinc finger CCCH domain-containing protein 33) (Table 4). The enrichment of zinc finger binding-like elements are higher in extremely tolerant genotypes compared to tolerant genotypes. This suggests a key role of ZnF in submergence tolerance although the molecular mechanism is not known yet. ZnF family TFs are known to be involved in important transcriptional regulation of plant responses to different abiotic stresses, such as drought, temperature, light, and salt (Wang et al., 2018). In rice, ZFP and C2H2 ZnF proteins are induced by a number of abiotic stresses including cold, drought, and salt (Jin et al., 2018). In our previous analysis, we identified high enrichment of zinc finger binding element-like elements associated with ZnF TFs as well as upregulation of a number of C2H2 and other ZnF proteins in the upregulated genes related to anaerobic metabolism in rice under anoxia (Lakshmanan et al., 2014). We also found upregulation of the expression of ZnF TF in wild-type rice under complete submergence (Mohanty et al., 2016). Besides, it has been reported that ZnF TF was upregulated in both rice and *Arabidopsis* in response to hypoxia/anoxia (Loreti et al., 2005; Lasanthi-Kudahettige et al., 2007; Pandey and Kim, 2012). In rice, a CCCH-type zinc finger protein was significantly induced by hypoxia/submergence stress indicating a key role during hypoxia/submergence stress in rice (Pandey and Kim, 2012). A B-box type zinc finger protein displayed significantly higher expression in soybean tolerant genotypes in response to flooding treatment compared to sensitive genotypes (Yu et al., 2019).

#### NAC Regulatory Module

High enrichment of DRE-like elements associated with NAC TFs is identified in the promoters of upregulated genes in all tolerant, highly tolerant, and extremely tolerant genotypes (Tables 1-3). This high enrichment can be associated with the activities of several NAC genes such as *Os11g0154500* [No apical meristem (NAM) protein domain-containing protein; NAC-domain-containing protein 90], *Os03g0815100* (Similar to OsNAC6 protein), *Os01g0884300* (NAC domain-containing protein 6), *Os07g0684800* (Similar to NAM/CUC2-like protein), and *Os07g0225300* (OsNAC3 protein) (Table 4). Members of NAC family TFs play a major role in regulating abiotic and biotic stresses in *Arabidopsis* and many other crops (Nakashima et al., 2012; Shao et al., 2015; Yuan et al., 2019a, b). In many cases, NAC TF is regulated through the ABA-dependent signal transduction pathway (Chen et al., 2014). In addition, there is evidence regarding the interaction of NAC and JA signaling pathway. Regulation of NAC through both ABA and JA to abiotic stress tolerance has been elucidated in *Arabidopsis* and other plants (Bu et al., 2008; Yoshii et al., 2010). It also maintains membrane integrity during abiotic stresses in *Arabidopsis* (Yong et al., 2019). It could be regulating germination and coleoptile elongation to maintain membrane integrity in cross-talk with other hormones such as ABA and JA. Moreover, reactive oxygen and nitrogen species (ROS and RNS) that accumulate during submergence/hypoxia activate a number of TFs including Heat Shock Factor (HSFs) and NAC families to control the homeostasis of the harmful molecules (Gonzali et al., 2015).

#### DOF Regulatory Module

Sequence motif “AAAAG/CTTTT”-element-like was identified in the promoters of all tolerant, highly tolerant, and extremely tolerant upregulated genes, which can be associated with DOF (DNA binding with one finger) TFs. These TFs are mainly plant-specific (Noguero et al., 2013) and are involved in the regulation of various processes in plant metabolism, seed germination, phytochrome response, and various developmental processes (Noguero et al., 2013; Wu et al., 2015). However, the biological function of this TF is not well studied in rice yet. Experiments on *OsDof3* suggested that it interacts with GAMYB to induce the expression of *RAmy1A* to facilitate GA signaling during the germination of rice seeds (Washio, 2003). In this analysis, DOF could be interacting with GA and other plant hormones at the initial stage of germination to activate the expression of stress-responsive genes to tolerate submergence during rice germination and coleoptile elongation.

#### HSF Regulatory Module

Enrichment of heat shock binding factor element-like in the promoter regions of upregulated genes of all tolerant, highly tolerant, and extremely tolerant genotypes could potentially be correlated with the upregulation of HSF genes such as *Os08g0471000* (Heat stress transcription factor B-4a, HSF20), *Os09g0526600* (Heat stress transcription factor B-2c, HSF 3), *Os09g0456800* (Heat stress transcription factor B-1), and *Os02g0232000* (Similar to Heat shock transcription factor 29, HSF 5) (Tables 1-4). HSFs are shown to be involved in heat stress and also other abiotic stresses (Nishizawa et al., 2008). Induction of a number of HSPs and HSTs in response to anoxia/hypoxia has been reported in *Arabidopsis* and rice (Loreti et al., 2005; Lasanthi-Kudahettige et al., 2007). HSF proteins play a major role in protecting cellular responses under stress conditions by preventing the misfolding and denaturation of proteins (Liberek et al., 2008) and also in the activation of HSP pathway during either anoxia or heat stress to cope with the production of ROS.

#### AS2/LBD Regulatory Module

Identification of a high enrichment of S2-binding-like elements in all tolerant, highly tolerant, and extremely tolerant genotypes proposes their possible association with Lateral organ boundaries domain (LBD) family TF AS2 (Tables 1-4). However, we did not see any expression of this gene in any of the tolerant genotypes. This TF plays a key role in different abiotic stress conditions. It is one of the hypoxia-induced TFs in *Arabidopsis* during flooding stress (Licausi et al., 2010). ARF7 and ARF19 TFs are known to regulate the expression of *LBD16* and *LBD29* for lateral organ development in *Arabidopsis* (Okushima et al., 2007). Recently, it has been shown that these TF genes could be regulated by auxin signaling under submergence (Wu and Yang, 2020), although the function is not known yet. This element could be present in the upregulated genes of all groups for lateral organ development later once the coleoptile reaches the surface of water for O_2_ availability.

#### E2F Regulatory Module

A moderate enrichment of E2F-binding site-like elements associated with E2F TFs was identified in the upregulated genes of the tolerant genotypes (Table 1). E2F TF plays a role in the regulation of cell division in rice coleoptile elongation under anoxia (Kosugi and Ohashi, 2002). The presence of E2F-binding site-like elements associated with E2F TFs was also detected in the promoters of upregulated genes under anoxia in rice coleoptile elongation and in wild-type rice demonstrating coleoptile elongation under anoxia (Mohanty et al., 2012, 2016).

#### TCP Regulatory Module

TCP binding site-like elements associated with TCP TF are enriched in the promoters of upregulated genes in tolerant and highly tolerant genotypes (Tables 1, 2). This TF plays a key role in cell growth, different hormone response pathways, and abiotic stress responses (Li et al., 2005; Danisman, 2016). The role of TCP in submergence tolerance is not known yet. However, in *Arabidopsis*, the activity of *AtTCP14* is necessary for seed germination and there is a functional relationship between this TF and GA (Tatematsu et al., 2008). Subsequently, both TCP14 and TCP15 were reported to regulate cell proliferation (Kieffer et al., 2011) and required for GA-dependent regulation of seed germination in *Arabidopsis* (Resentini et al., 2015). Identification of TCP-binding sites in tolerant and highly tolerant genotypes suggests their roles in initial cell proliferation of coleoptile during germination under submergence in rice.

#### HD-ZIP (ATHB4) Regulatory Module

Promoter analysis identified the presence of HD-ZIP binding site-like elements associated with HD-ZIP TFs in the promoters of all upregulated genes of all tolerant, highly tolerant, and extremely tolerant genotypes (Tables 1-3). The enrichment of this element is most probably associated with the activities of a number of HD-ZIP genes such as *Os06g014040* (Homeobox protein knotted-1-like 10), *Os05g0129700* (Homeobox-leucine zipper protein HOX28), *Os03g0198600* (Homeodomain-leucine zipper transcription factor), *Os06g0140700* (Homeobox-leucine zipper protein HOX2), *Os03g0188900* (Homeobox-leucine zipper protein HOX13), and *Os09g0528200* (Similar to Homeobox-leucine zipper protein HOX6) (Table 4). HD-ZIP is involved in the regulation of many developmental processes and response to different abiotic stresses in many plants (Annapurna et al., 2016; Tang et al., 2019; Zhao et al., 2019). However, the role of HD-ZIP in response to submergence stress is not known yet. In *Arabidopsis* seedlings, *AtHB4* gene was shown to regulate both shade avoidance and hormone-mediated development, particularly BR (Sorin et al., 2009). *AtHB4* acts downstream of P1F1 and was involved in the activation of hypocotyl cell wall composition and elongation in response to short day plants (Capella et al., 2015). The presence of this binding site in highly tolerant and extremely tolerant genotypes could be playing a role in coleoptile elongation by mediating the BR signaling pathway.

#### ARR-B Regulatory Module

ARR-B binding element-like associated with type-B ARRs was identified among upregulated genes in all tolerant, highly tolerant, and extremely tolerant genotypes (Tables 1-3). Type-B ARRs such as ARR1-ARR2, ARR10-ARR14, and ARR18-ARR21 play a key role as positive regulators of cytokinin signaling (Argyros et al., 2008). Besides, they also play an important role in the downregulation of ABA activity in response to cytokinin. Moreover, the binding sites of ARR-B TFs are known to be associated with metabolism of BR, BR signaling, and transcriptional regulation by TFs such as BZR2; BEH1, 2, 3, and 4; BIM1; and MYB30 (Zubo and Schaller, 2020). Genes that belong to ARR-B are also involved in the induction of GA biosynthesis and the reduction of GA perception (Marín-de la Rosa et al., 2015). These binding sites were identified in the genes associated with ethylene biosynthesis and signal transduction. Since genes in all genotypes possess these binding sites, it suggests that they could be playing a key role together with other hormones to regulate the elongation and germination of coleoptile in response to submergence tolerance.

#### MADS-box (AGL) Regulatory Module

High percentage of CArG box-binding site-like elements corresponding to MADS box (AGL) TF were identified in the upregulated genes of all tolerant, highly tolerant, and extremely tolerant genotypes (Tables 1-3), and this could be due to the induction of *Os04g0580700* (MADS-box transcription factor 17) (Table 4). MADS box (AGL) TFs are key regulators of many developmental processes in plants (Smaczniak et al., 2012). However, their role in seed germination is unknown except for a few MADS-box genes such as *AGL25*, *AGL67*, and *AGL 21* (Chiang et al., 2009; Bassel et al., 2011; Yu et al., 2017). *AGL25* is reported to be involved in temperature-dependent seed germination by inducing the GA biosynthetic pathway and the ABA catabolic pathway, whereas *AGL67* and *AGL21* act as negative regulators of seed germination. AGL21 incorporates both hormone signal and environmental signal to ABA signaling by balancing ABI5 to prevent germination under adverse conditions (Yu et al., 2017).

#### BES/BZR Regulatory Module

Interestingly, E-box-like elements associated with BES/BZR TFs involved in Brassinosteroid (BR) signaling pathway were identified only in the upregulated genes of highly tolerant and extremely tolerant genotypes that had longer coleoptile elongation in response to submergence (Tables 2, 3). BR is one of the most important plant steroidal hormones that regulate a wide range of plant growth, development including cell elongation and seed germination, and responses to biotic and abiotic stresses (Guo et al., 2013; Tong et al., 2014; Zhang et al., 2014; Li et al., 2016; Ahammed et al., 2020). It has also been shown earlier that BRs can increase coleoptile length in rice (Yamamuro et al., 2000). However, the molecular mechanism of BR that controls the germination and coleoptile elongation of rice seeds is not well known yet. High level of BR inactivates BIN2 and activates dephosphorylation of TFs BZR1 and BZR2/BES1 by protein phosphatase for their accumulation in the nucleus to increase their DNA-binding activity for the expression of BR-responsive genes (Wang et al., 2012). BZR1 and BZR2/BES1 regulate a large number of structural and metabolic genes as well as cell wall biogenesis and regulatory genes (Yu et al., 2011). Gene expression profiles have also revealed the induction of the expression of many cell wall extension and loosening enzymes and expansins (Guo et al., 2009). Several studies have reported that rice has a well-preserved BR signaling pathway like *Arabidopsis* (Li et al., 2009; Tong and Chu, 2012; Tong et al., 2014). It also regulates developmental processes through a cross-talk interaction with other signaling pathways. In lowland rice plants, both BRs and GA act antagonistically in response to submergence tolerance (Schmitz et al., 2013). They have indicated that BR induces GA catabolic genes and also DELLA proteins to limit GA level during submergence, and the cross-talk between BR and GA depends on tissues and hormone levels in rice (Tong et al., 2014). Although ABA and BR signaling are known to be antagonist of each other, recently it has been reported that both BR and ABA co-regulate to enhance seed germination in *Arabidopsis*. BES1, an important TF of the BR signaling pathway, assists in seed germination by weakening ABA signaling pathway induction by interfering the transcriptional activity of ABI5 (Zhao et al., 2019).

BR also activates different stress adaptive signaling pathways by directly or indirectly regulating different stress-responsive TFs such as bZIP, MYB, WRKY, NAC, DREB, etc. through BIN2 and key BZR1/BES1 TFs (Sharma et al., 2017). Genetic analysis shows that BZR1 and PIF, a bHLH TF, directly interact and promote cell elongation and etiolation (Oh et al., 2012). Moreover, BZR1 and PIF4 along with ARF6 promote genes that are involved in cell expansion (Oh et al., 2014). Both E-box motifs and G-box (CACGTG) motifs are associated with TF BZR1/2 and PIFs, and these are highly enriched in ARF6 binding regions. BEE2 is a bHLH TF, which is shown to be involved in the regulation of cell elongation by BR (Friedrichsen et al., 2002). BEE2 also interacts with ARF6 like PIF6 to promote cell elongation (Oh et al., 2014). These findings suggest a key role of BR-responsive TFs in cell elongation.

#### bHLH Regulatory Module

E-box-like/G-box-like elements associated with bHLH (Gr. III and VII) including PIFs were identified in both highly tolerant and extremely tolerant genotypes (Tables 2, 3). Interestingly, a set of E-box-like and BBRE-element-like/G-box-like elements associated with TFs such as BEE2, BIM1, BIM3, BAM8, and BEH4 that belong to bHLH were identified only in the upregulated genes of the two extremely tolerant genotypes. These elements could be associated with the induction of different bHLH TF genes such as *Os01g0773800* (bHLH protein 185), *Os03g0188400* (bHLH protein), *Os07g0628500* (bHLH dimerization region bHLH domain-containing protein), *Os03g0135700* (bHLH transcription factor), and *Os07g0143200* (Phytochrome-interacting bHLH factor) (Table 4). bHLH TFs are involved in the regulation of many cellular processes (Zhao et al., 2020) such as seed germination (Penfield et al., 2005), light signaling (Leivar et al., 2008), hormone signaling (Fernández-Calvo et al., 2011), responses to wounding, drought, salt, and low temperature (Sun et al., 2018). They also play a key role in BR-responsive gene expression to support coleoptile elongation, which has already been discussed earlier.

Phytochrome-interacting factors (PIFs) are bHLH TFs. Besides the bHLH domain, they have active phytochrome A/B binding domains. They are involved in various physiological processes such as seed germination, photomorphogenesis, shade responses, flowering time, and leaf senescence (Leivar and Quail, 2011; Casal, 2013; Sakuraba et al., 2014). Besides, PIFs promote cell elongation under low Red:far Red light conditions by inducing the transcription of genes related to growth (Paik et al., 2017). Involvement of PIFs in signaling responses (GH3, IAA, and ARF), cell wall modification, and elongation has been studied (Zhang et al., 2013; Pfeiffer et al., 2014). In addition, they are involved in a variety of hormone-response pathways such as GA, BR, JA, ethylene, and nitric oxide (Mazzella et al., 2014; Paik et al., 2017). However, the study of phototropism of rice coleoptile under submergence is not known yet. Interestingly, it was suggested that submerged coleoptiles exhibited only a slight R-induced growth inhibition (Pjon and Furuya, 1974). Studies show that they are less phototropic compared to other gramineae coleoptiles such as maize and oats (Neumann and Iino, 1997). Experiments on the *coleoptile photomorphogenesis 1* (*cpm1*) mutant suggest that this gene has a role in phytochrome-mediated inhibition of coleoptile growth. Later studies on phototropism rice coleoptile demonstrates that auxin is involved in this process (Haga et al., 2005).

### Identification of Putative *cis*-Elements in the Upregulated Genes of Intermediately Tolerant Diverse Rice Genotypes in Response to Submergence Tolerance

To compare and validate the identification of *cis*-elements in tolerant, highly tolerant, and extremely tolerant genotype groups, upregulated genes in the intermediate group, which has two highly tolerant genotypes, F291 and F274-2a, were also analyzed (Table 5). The analysis results identified highly enriched significant *cis*-elements that are associated with MYB, bZIP, ABI5, different groups of AP2/ERF, AP2/B3, EIL, ARF, WRKY, ZnF, NAC, DOF, HSF, AS2, TCP, ARR-B, MADS box, GATA, BES/BZR, bHLH, and BPC2 (Table 5). Interestingly, the analysis results for the intermediate group are exactly similar to the identification of *cis*-elements and their associated TFs in highly tolerant genotypes (Table 2). In addition, it is slightly different in terms of promoter architecture content from the analysis that was performed for extremely tolerant genotypes (Table 3). Hence, the analysis results for both intermediately tolerant genotypes and highly tolerant genotypes suggest that they have a common gene regulatory mechanism that allows longer coleoptile elongation of rice seeds during germination, in response to submergence tolerance.

**TABLE 5:** Potential putative *cis*-elements identified in the promoters of upregulated genes in two intermediately tolerant genotypes (F291 and F274-2a).

***Cis*-elements**	**Motifs**	**Associated TFs**	% (TIC), *E*-value*
AT-hook/PE1-like	AGAAAAATG	MYB (PF1)	58 (14.34), 2e-004
GT-element-like	TTTGTTCA CATGTGTG TTTACTCT CTCTTAAA	MYB (GT-3) MYB (GT-3a) MYB (GT1/GT2) MYB (GT1/GT2)	71 (13.29), 2e-004 58 (12.27), 2e-004 50 (12.92), 1e-004 50 (12.19), 0e+000
GARE-like	TTTGTTCA	MYB (R1, R2R3)	71 (13.29), 2e-004
MYB-box-like	CCACCATG TTTGTTCA GCAAGGTG GGTTCGTC GATGGTATT	MYB (R2R3) MYB (R2R3) MYB (R2R3) MYB (R2R3) MYB (R2R3)	79 (11.60), 3e-005 71 (13.29), 2e-004 67 (12.65), 3e-005 58 (11.20), 6e-005 54 (13.12), 2e-004
MYB-box related-like	GCCACCAT	MYB-like	63 (12.88), 3e-004
As-1/ocs-like	GCAAGGTG AAGTTTGA CATGTGTG	bZIP (Gr. D, I, S) bZIP (Gr. D, I, S) bZIP (Gr. D, I, S)	67 (12.65), 3e-005 63 (12.83), 1e-004 58 (12.27), 2e-004
GCN4 motif	TTTGTTCA AGAAAGTG	bZIP (RISBZ1, Gr. G) bZIP (RISBZ1, Gr. G)	71 (13.29), 2e-004 54 (13.04), 6e-005
G-box-like	GCAAGGTG CATGTGTG	bZIP (Gr. G) (GBF 3, 5) bZIP (Gr. G) (GBF 3, 5, 6)	67 (12.65), 3e-005 58 (12.27), 2e-004
ABRE-like (DPBF binding site-like)	GCAAGGTG CATGTGTG	bZIP (DPBF-3) bZIP (DPBF-3) (Opaque-2)	67 (12.65), 3e-005 58 (12.27), 2e-004
ABRE-like	GCAAGGTG CATGTGTG	ABI5 (bZIP) ABI5 (bZIP)	67 (12.65), 3e-005 58 (12.27), 2e-004
ABRE-like	CATGTGTG	ABF2 (bZIP)	58 (12.27), 2e-004
GCC-box-like	GCCACCAT GCCGGAAA	ERF (I, IV, VII, X) ERF (I, IV, VII, X)	63 (12.88), 3e-004 50 (12.53), 3e-004
ERE-like	GCAAGGTG GCCACCAT GGTTCGTC GCCGGAAA	ERF/RAP2.1, RAP2.4, RAP2.9, RAP2.10 ERF/RAP2.3, RAP2.6 (Gr. III) ERF/RAP2.3, RAP2.6 (Gr. III) RAP2.11(Gr. III)	67 (12.65), 3e-005 63 (12.88), 3e-004 58 (11.20), 6e-005 50 (12.53), 3e-004
CRT/DRE-like	GCCACCAT GCCGGAAA	ERF (Gr., III, IV) ERF (Gr., III, IV)	63 (12.88), 3e-004 50 (12.53), 3e-004
JA response element-like	AAGTTTGA	ERF (Gr. VI, VIII, IX) ERF (Gr. VI, VIII, IX)	63 (12.83), 1e-004 69 (13.75), 4e-004
ABRE-like	CATGTGTG	FUS3, (Similar to VP1/ABI3-like proteins) FUS3, (Similar to VP1/ABI3-like proteins)	58 (12.27), 2e-004 69 (11.61), 5e-005 50 (13.95), 3e-005
ABRE-like	AGAAAGTG AGAAAAATG	AP2/B3 (Gr. II) Related to RAV2 AP2/B3 (Gr. II) Related to RAV2	54 (13.04), 6e-005 58 (14.34), 2e-004
ABR-binding site-like	GCGGGAGA	AP2-like ABA repressor 1 (ABR1)	54 (11.84), 9e-005
Ethylene-insensitive 3 binding site-like	TTTGTTCA GCCGGAAA	EIL3 (EIN3) EIN2	71 (13.29), 2e-004 50 (12.53), 3e-004
Aux-RE-like	TTTGTTCA AGAAAAATG	ARF1 ARF16	71 (13.29), 2e-004 58 (14.34), 2e-004
W-box-like	TTTGTTCA	WRKY (Gr. I, IIa, IIc, III)	71 (13.29), 2e-004
Zinc-finger-binding site-like	GCAAGGTG GGTTCGTC AGAAAGTG GATGCGATT GCCGGAAA	ZnF (C2H2-type) ZnF (C2H2) ZnF (C2H2) ZnF (CCCH-type) ZnF (PhD) ZnF (PhD)	71 (13.29), 2e-004 58 (11.20), 6e-005 54 (13.04), 6e-005 54 (13.12), 2e-004 50 (12.53), 3e-004
IDD binding site-like	TTTGTTCA AGAAAAATG	IDD 2, 4, 5, 7 (ZnF) IDD 4, 5 (ZnF)	71 (13.29), 2e-004 58 (14.34), 2e-004
DRE-like	CCACCATG GGTTCGTC CATGTGTG AGAAAAATG AGAAAGTG CTCTTAAA AGCCGTAG	NAC (16, 92) NAC (46, 55, 58) NAP (NAC-like, activated by AP_3_/P_1_) NAM NAC (57, 71, 83, 103) CUC1, CUC2, CUC3 NAC (5, 11) NAC (5, 11, 20, 28, 45, 50, 58, 62, 71, 75, 96) VND (2, 3, 4, 6) CUC1, CUC2, CUC3 NAC (5, 62) NAC (58, 80, 87, 92) VND1, CUC1, CUC2, CUC3	79 (11.60), 3e-005 58 (11.20), 6e-005 58 (12.27), 2e-004 58 (14.34), 2e-004 54 (13.04), 6e-005 50 (12.19), 0e+000 50 (12.65), 8e-005
AAAAG/CTTTT-element-like	ATCCCTTT AGAAAAATG AGAAAGTG	DOF (PBF) DOF-type zinc finger DOF (DOF 4) DOF (DOF 5.7)	67 (11.52), 1e-004 58 (14.34), 2e-004 54 (13.04), 6e-005
Heat shock binding factor element-like	CTCCCCCT AGAAAAATG AGAAAGTG	HSF (HSFB2A) HSF (HSFB2A) HSF (HSFB2A)	67 (12.51), 2e-004 58 (14.34), 2e-004 54 (13.04), 6e-005
S2-binding site-like	CTCCCCCT GCCACCAT GCCGGAAA	AS2 (LBD13) AS2 (LBD23) AS2 (LBD2, 13, 16)	67 (12.51), 2e-004 63 (12.88), 3e-004 50 (12.53), 3e-004
TCP binding site-like	GGTTCTTC	TCP (3, 20)	58 (11.20), 6e-005
ARR14-binding element-like	GATGCGATT	ARR-B (ARR14)	54 (13.12), 2e-004
CArG box-binding site-like	CCACCATG GCCACCAT AGAAAAATG AGAAAGTG TTTACTCT	MADS (AGL 42, 55) MADS (AGL 55) MADS box (AGL 6, 15, 16) MADS box (AGL 6, 15, 16) MADS (AGL 16)	79 (11.60), 3e-005 63 (12.88), 3e-004 58 (14.34), 2e-004 54 (13.04), 6e-005 50 (12.92), 1e-004
GATA binding site-like	TCTTCCAT GCAAGGTG	GATA1 GATA1	67 (12.72), 3e-005 67 (12.65), 3e-005
E-box-like	CATGTGTG	BES/BZR (BES1/BZR1 homologue 2, 3, 4)	58 (12.27), 2e-004
E-box-like/G-box-like	GAAGTAAC CATGTGTG AGCCGTAG	bHLH (Gr. III, VII) bHLH (Gr. III, VII) bHLH (Gr. III, VII)	63 (11.53), 1e-004 58 (12.27), 2e-004 50 (12.65), 8e-005
BPC-binding site-like	AGAAAAATG AGAAAGTG	BPC (BPC1) BPC (BPC1)	58 (14.34), 2e-004 54 (13.04), 6e-005

**%** = percent occurrence among all upregulated genes, TIC = total information content of homology, *E*-value* = *E*-value of homology with promoter database entry.

Similarly, to validate the identification results, analysis was performed for a set of upregulated genes that have the moderate genotype Nipponbare and highly tolerant genotype F274-2a. The analysis results identified *cis*-elements that are associated with TFs such as MYB, bZIP, ERF, ABI3, AP2/B3, ARF, ZnF, NAC, HSF, AS2, TCP, ARR-B, MADS box, GATA, DBP1, and TBP (Table 6). Remarkably, the potential TFs that were identified also show similar promoter architecture with results obtained for tolerant genotypes (Table 1). The analysis results also did not identify specific *cis*-elements that are associated with BES/BZR and bHLH TFs present in highly tolerant and extremely tolerant genotypes (Tables 2, 3).

**TABLE 6:** Potential putative *cis*-elements identified in the promoters of upregulated genes in a group that has one tolerant genotype and one highly tolerant genotype (Nipponbare and F274-2a).

***Cis*-elements**	**Motifs**	**Associated TFs**	% (TIC), *E*-value*
AT-hook/PE1-like	TTTTTTCA AAAAAAATA GTTTTTTTT	MYB (PF1) MYB (PF1) MYB (PF1)	55 (13.88), 3e-004 50 (15.40), 1e-004 50 (15.39), 4e-004
GT-element-like	TGGTTTGT GGGGAAAA AAAATATCT	MYB (GT-3) MYB (GT-1/GT-3) MYB (GT-1)	69 (11.62), 1e-004 64 (12.14), 4e-004 58 (13.64), 2e-004
Pyrimidine box-like	TTTTTTCA	MYB (R1, R2R3)	55 (13.88), 3e-004
GARE-like	TGGTTTGT	MYB (R1, R2R3)	69 (11.62), 1e-004
MYB-box-like	TGGTTTGT AAAACCAA AACCATGC	MYB (R2R3) MYB (R2R3) MYB (R2R3)	69 (11.62), 1e-004 64 (12.42), 2e-004 57(11.65), 4e-004
As-1/ocs-like	TTTTTTCA CTGCAGGC	bZIP (Gr. D, I, S) bZIP (Gr. D, I, S)	55 (13.88), 3e-004 57 (11.69), 5e-004
GCN4 motif	TGGTTTGT	bZIP (RISBZ1, Gr. G)	69 (11.62), 1e-004
GCC-box-like	CGCCGCCGC	ERF (I, IV, VII, X) ERF (I, IV, VII, X)	52 (15.43), 7e-004
ERE-like	CTGCCGGC CTGCAGGC	ERF/RAP2.1, RAP2.2, RAP2.3, RAP2.6, RAP2.10, RAP2.11, RAP2.12 (Gr. III) ERF/RAP2.2, RAP2.12 (Gr. VII)	57 (11.69), 5e-004 57 (11.69), 5e-004
CRT/DRE-like	CTGCCGGC	ERF (Gr., III, IV)	57 (11.69), 5e-004
ABRE-like	AACCATGC	ABI3/V1P1 (B3 domain)	57(11.65), 4e-004
ABRE-like		AP2/B3 (Gr. II) Related to RAV2	56 (12.79), 2e-004
ABR-binding site-like	CTGCCGGC	AP2-like ABA repressor 1 (ABR1)	57 (11.69), 5e-004
Aux-RE-like	TGGTTTGT	ARF1	69 (11.62), 1e-004
Zing finger binding site-like	GGGGAAAA TATATGTA AAAATATCT	ZnF (C2H2-type) ZnF (C2H2-type) ZnF (C2H2-type)	64 (12.14), 4e-004 62 (12.72), 1e-004 58 (13.64), 2e-004
DRE-like	ACTCTTCC AACCATGC	NAC 20 NAC (4, 20, 38, 50, 57, 58, 70, 83, 103) SND3, CUC1, CUC2, CUC3	69 (11.29), 3e-004 57(11.65), 4e-004
Heat shock binding factor element-like	ACTCCCCC GGGGAAAA	HSF (HSFB2A) HSF(HSFB2A)	69 (11.29), 3e-004 64 (12.14), 4e-004
S2-binding site-like	ACTCCCCC CTCCTCCT	AS2 (LBD13) AS2 (LBD13)	69 (11.29), 3e-004 57 (13.60), 3e-005
CArG box-binding site-like	ACTCTTCC GGGGAAAA	MADS-box (AG 16) MADS-box (AG 15, 16)	69 (11.29), 3e-004 64 (12.14), 4e-004
GATA binding site-like	TGGTTTGT	GATA 1	69 (11.62), 1e-004
DBP-binding site-like	AAAATTAT GAAATATT	DBP1 DBP1	65 (12.40), 3e-004 51 (12.80), 3e-004
TATA-box-like	GAAATATT	TBP	51 (12.80), 3e-004

**%** = percent occurrence among all upregulated genes, TIC = total information content of homology, *E*-value * = *E*-value of homology with promoter database entry.

## Discussion

The global climate variation causes extreme weather changes and often causes severe rain, which is harmful for rice seed germination and seedling growth. Although rice has the unique ability to tolerate such conditions, different genotypes of rice show different degrees of tolerance to submergence by elongating their coleoptiles. Hence, it is essential to understand the regulatory mechanism that helps in tolerating such adverse conditions. It is a complex process and involves interactive mechanisms of both metabolic and transcriptional regulation and hormonal signaling. A number of studies show that different types of hormones mainly activate transcriptional regulation that enables different plant metabolic processes to tolerate any adverse conditions due to climate change (Nemhauser et al., 2006). During rice germination and coleoptile elongation under submergence, although ethylene and ERF factors are known to be involved, the role of other TFs and hormonal signaling is not well understood yet. Therefore, the promoter architecture of upregulated genes of tolerant genotypes with different rates of coleoptile elongation could give some indication regarding the *cis*-elements content and their association with specific TFs. This information would support in providing a regulatory role of potential TFs and different hormonal signaling pathways involved in the transcriptional regulation of submergence tolerance mechanism. In this study, promoter architecture was analyzed for different sets of common genes associated with different degrees of submergence tolerance such as tolerant genotypes (Nipponbare, two recombinant inbred lines F291 and F274-2a, and two natural genotypes 8391 and 8753), highly tolerant genotypes with longer coleoptile elongation (F291, F274-2a, 8391, and 8753), and extremely tolerant natural genotypes with the longest coleoptile elongation (8391 and 8753). The promoter architecture analysis of upregulated genes for tolerant genotypes including *O. sativa japonica* identified the presence of putative *cis*-elements that are associated with TFs such as MYB, bZIP, AP2/ERF, ARF, EIN3, ABI3, ABR1,WRKY, ZnF, MADS-box, NAC, AS2, DOF, HD-ZIP, E2F, ARR-B, and HSF (Table 1). For the verification of these findings, promoters of the upregulated genes of a group that has tolerant genotypes, Nipponbare and F274-2a, were analyzed. The analysis identified most of the *cis*-elements that are associated with TFs identified in tolerant genotypes (Table 6). Interestingly, in addition to these TFs, there was identification of more specific binding sites associated with different specific TFs such as ABRE-binding site associated with bZIP, ABI5, ABF2, and bZIP (DPBF3); HY-5 binding site-like associated with HY5; G-box-like associated with GBF3, 5, and 6; E-box-like associated with BES/BZR TF; and E-box/G-box-like elements associated with bHLH (PIF7) in highly tolerant genotypes (Table 2). Moreover, the promoter architecture of the extremely tolerant genotypes (longest coleoptile elongation) is quite fascinating. They contain binding sites that are present in both tolerant and highly tolerant genotypes as well as higher enrichment of more binding sites such as ABRE-like associated with both ABF1 and ABF2 (bZIP), E-box-like/G-box-like and BBRE-element-like/G-box-like associated with bHLH, BEE2(bHLH), and BAM8 (bHLH) involved in BR-mediated signaling (Table 3). These binding sites are completely absent in the tolerant genotypes and less enriched or absent in highly tolerant genotypes. To support these findings, an additional promoter architecture analysis was performed by taking an intermediate genotype group that has two highly tolerant genotypes, F291 and F274-2a. The putative *cis*-element analysis of this group also identified less enrichment or complete absence of those specific binding sites that are present in the extremely tolerant genotypes (Table 5). These findings clearly show that the promoter architecture varies from genotype to genotype and the difference in tolerance mechanism and coleoptile elongation could be dependent on the presence of specific binding elements that are associated with specific transcriptional regulation by TFs in the promoter of genes upregulated during submergence.

**FIGURE 2:**
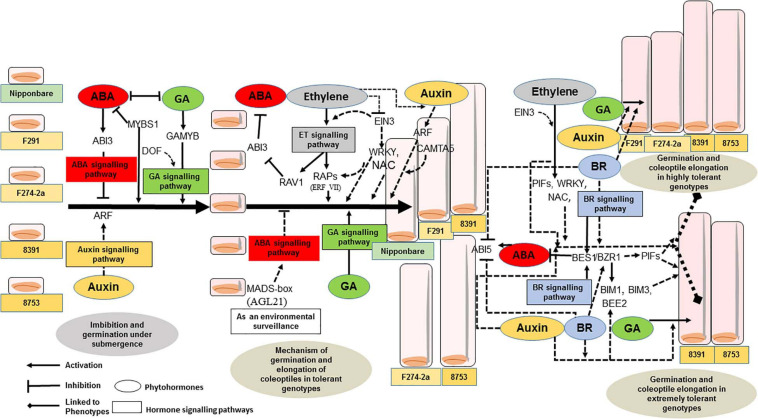
Hypothetical model representing transcriptional regulatory mechanisms leading to differences in germination and coleoptile elongation of diverse rice genotypes in response to submergence tolerance. TFs acting as activators/repressors along with hormonal signaling induce the synthesis of cell building blocks for elongation of coleoptiles in response to submergence in rice genotypes from diverse backgrounds. The differences in the length of coleoptiles could be due to altered transcriptional regulatory mechanisms involving bHLH and BES/BZR TFs together with other TFs and a cross-talk between BR and other signaling pathways. The dashed lines represent the hypothesis predicted from the gene expression data of tolerant, highly tolerant, and extremely tolerant genotypes that exhibit differences in coleoptile elongation in response to submergence tolerance.

Based on the promoter architecture of genes upregulated in all the three groups of different genotypes, the results suggest that there is involvement of both MYBs such as MYB (R1, R2R3) and MYB (R2R3) during the initial stage, i.e., imbibition and germination where both hormones ABA and GA play a key role. Upon imbibition of rice seed, the GA content gradually increases and breaks dormancy by inducing the secretion of hydrolytic enzymes and the endogenous ABA level decreases rapidly due to the induction of ABA catabolic genes (Figure 2). GAMYB is a GA-responsive R1, R2R3 TF that induces the expression of *α-amylase gene* (*RAmy3D*) in the aleurone layer for starch degradation to provide substrates for the germination and coleoptile elongation of rice seeds (Perata et al., 1997) under submergence. During this process, DOF TF interacts with GAMYB for induction of the expression of α*-amylase* to enable GA signaling (Zou et al., 2008; Figure 2). The high enrichment of MYB-box-like elements associated with MYB R2R3 TF and ABA signaling suggest that these TFs that are involved in seed maturation might be stored in rice seeds to initiate initial germination process during the early stage of germination (Sreenivasulu et al., 2008). High enrichment of ERF-VII TFs including RAP2.2, RAP2.3, RAP2.6, RAP2.10, and RAV1 shows their involvement and the importance of the hormone ethylene in germination and coleoptile elongation of rice seeds in response to submergence. The gaseous hormone ethylene is required for the transport of sucrose to the coleoptile (Ishizawa and Esashi, 1988). This is again supported by the higher expression of two ERF genes such as *LOC_Os01g21120* in all genotypes [higher expression in tolerant genotypes compared to sensitive genotype (IR64) and higher expression of *LOC_Os07g47790* in the tolerant genotypes] (Hsu and Tung, 2017). The role of ethylene in coleoptile elongation is also supported by the identification of EIN3-binding site elements in all tolerant, highly tolerant, and extremely tolerant genotypes (Tables 1 – 3). EIN3 becomes active in the dark and regulates the ethylene signaling pathway to transcribe ethylene-responsive genes. Since this TF has a unique role in regulating multiple transcriptional regulation, it could also be involved in feedback regulation by ethylene and regulation of other hormones and TFs (Dolgikh et al., 2019; Figure 2). Besides, high enrichment of ERE-like elements associated with ERF TF (Gr. III) such as RAP2.2, RAP2.3, RAP2.6, and RAP2.10 suggests their roles in fermentation and sugar metabolism in response to submergence (Tables 1-3). Identification of RAV-like elements associated with RAV1 implies their role in repressing the ABA signaling pathway by inhibiting the expression of ABI3, ABI4, and ABI5 by binding to the 5′−CAACA−3′ *cis*-element in their promoters (Feng et al., 2014; Figure 2). The enrichment of binding sites associated with ERF TFs including GCC-box-like elements associated with ERF (I, IV, VII, and X) was highest in tolerant genotypes and less enriched in the extremely tolerant genotypes. High enrichment of RAP2.3, RAP2.6, RAP2.10, and ERF (Gr. VII) could be associated with a common role of ethylene in all genotypes. The enrichment of ARF binding sites associated with TF ARF and the expression of ARF gene suggest the role of auxin in an auxin-dependent elongation of rice coleoptile through the interaction with ethylene (Ishizawa and Esashi, 1984; Breviario et al., 1992). Interaction of both hormones also inhibited root elongation in rice seedlings (Qin et al., 2017) and plays an important role in cell division and regulation of carbohydrate metabolism (Wu and Yang, 2020). Auxin upregulates GA biosynthesis genes and regulates the expression of GA metabolism genes through the action of Aux/IAA and ARF (Frigerio et al., 2006). Moreover, the co-regulation of both ethylene and auxin happens at the level of transcription as well as transport response. Besides AuxRE-like elements, there was identification of CAMTA5 binding site-like elements associated with bZIP (CAMTA5) TF. This TF could be involved in regulating auxin transport and homeostasis (Galon et al., 2010).

It is known that bZIP 11 (Gr. S1) is induced by sucrose and can regulate sugar metabolism. It is most probably involved in adaptations to carbon starvation in *Arabidopsis* (Ma et al., 2011). bZIP also activates genes involved in trehalose metabolism and amino acid metabolism in response to stress (Ma et al., 2011; Weiste and Dröge-Laser, 2014). In rice, *OsTPP7* encodes trehalose-6-phosphate phosphatase and is involved in the induction of starch mobilization during germination and coleoptile elongation (Kretzschmar et al., 2015). However, this gene was found to be upregulated in Nipponbare and the two RIL genotypes, F291 and F274-2a, but downregulated in two natural genotypes, and that could be due to structural variation (Hsu and Tung, 2017). Identification of as-1/ocs-like elements associated with bZIP (Gr. D, I, S) in tolerant, highly tolerant, and extremely tolerant genotypes propose their possible role in carbon and amino acid metabolism during coleoptile elongation in response to submergence. The majority of the bZIP TFs play a key role in ABA signaling pathways. G-box-related *cis*-elements associated with bZIPs are found in auxin-induced promoters and function as modulators of auxin-induced transcription in *Arabidopsis* (Weiste and Dröge-Laser, 2014). High enrichment of ABRE-like and G-box-like elements associated with bZIP TF could probably be involved in auxin-induced transcription for the longer elongation of coleoptiles in response to submergence in highly tolerant and extremely tolerant genotypes (Figure 2).

High enrichment of MYB-box-like elements associated with MYB R2R3 TF in the promoter regions of upregulated genes in all groups of genotypes indicates the involvement of pre-existing ABA not only at the beginning of germination but also at a later stage of germination through activation–inhibition machinery (Tables 1-3). It has been reported that ethylene–ABA interaction inhibits root growth in rice seedlings (Ma et al., 2014). In the dark, ethylene plays a double role in rice: stimulates coleoptile elongation and inhibits root growth (Ma et al., 2010, 2013; Yang et al., 2015), which is different from the response of ethylene in *Arabidopsis* (Bleecker and Kende, 2000). Moreover, it is motivating to find the presence of E-box-like/G-box-like elements associated with bHLH TFs such as PIF3 and PIF7 and BES/BZR TF in the promoters of upregulated genes in the highly tolerant and extremely tolerant genotypes (Tables 2, 3). Among them, the enrichment of the binding sites are much higher in the extremely tolerant natural genotypes. bHLH TFs are major players in phytochrome signal transduction and expression of BR-responsive genes (Duek and Fankhauser, 2005; Serna, 2007). PIFs and the hormone BR could be contributing in the regulation of longer cell elongation of rice coleoptiles under submergence (Paik et al., 2017) compared to moderate elongation in the *O. sativa japonica* genotype (Figure 2). Among the highly tolerant and extremely tolerant genotypes, E-box-like/G-box-like elements and BBRE-element-like/G-box-like element are more enriched in the extremely tolerant natural genotypes compared to the two recombinant inbred lines (RILs; F291 and F274-2a) derived from a cross between Nipponbare and IR64 (Tables 2, 3). BZR1 and PIF4 together with ARF4 induce genes that are involved in cell expansion (Oh et al., 2014). Moreover, BEE2 also regulates cell elongation by BR and interacts with ARF6 (Oh et al., 2014). These data indicate a potential involvement of BEE2, PIFs (bHLH), and BR-mediated signaling pathways contributing to the phenotypes of the two natural genotypes in regulating the highly elongated coleoptile growth compared to the RILs (Figure 2). Additionally, BR is reported to stimulate seed germination and inactivate the negative effect of ABA on seed germination by a negative feedback mechanism that modulates ABA signaling (Steber and McCourt, 2001; Xi et al., 2010).

During submergence/flooding stress, rice coleoptiles also suffer from low osmotic stress in addition to hypoxia/anoxia stress. A number of TFs such as AREB/ABFs are known to regulate ABA signaling during such stress conditions (Yoshida et al., 2010). In this promoter architecture analysis, identification of ABRE-like elements associated with ABF1 and ABF2 TFs could be involved in osmotic stress tolerance in the highly tolerant and extremely tolerant genotypes where the coleoptile elongation is higher compared to the moderate genotype *O. sativa japonica*. Among these two groups, there is high enrichment of ABF1 and ABF2 associated motifs in the extremely tolerant genotypes compared to highly tolerant genotypes (Tables 2, 3). As these TFs are associated with ABA signaling, the endogenous ABA level could be acting as an on–off switch having a spatiotemporal regulation to activate/repress other TFs and hormonal signaling pathways during the germination and coleoptile elongation under submergence (Figure 2). The enrichment of ABRE-like elements associated with both ABI3 and ABI5 TFs in all five tolerant genotypes is complicated as both are positive regulators of ABA signaling and involved in the arrest of seed germination (Lopez-Molina et al., 2002). They have shown that ABI5 is a bZIP TF, and it acts downstream of ABI3 to inhibit germination. A MADS-box TF AGL21 positively regulates the expression of ABI5 and responds to a number of environmental stresses and plant hormones during seed germination (Yu et al., 2017). It is reported that it could be acting as a surveillance integrator to incorporate external environmental signals and endogenous hormonal signals to ABA signaling for the regulation of seed germination and early post-germination growth. In this analysis, identification of putative *cis*-elements associated with ABI3 and ABI5 and high enrichment of MADS-box (AGL) TFs indicate a probable role of AGL as an environmental surveillance integrator to safeguard seed germination process by inducing ABA signaling through ABI5 TF (Figure 2). Although the putative *cis*-element associated with ABI3 is present in all groups, the elements associated with ABI5 are more enriched in highly tolerant and extremely tolerant genotypes. Also, during seed germination in *Arabidopsis*, JA enhances ABA activation to inhibit seed germination through JAZ repressors of the JA signaling pathway by regulating ABI3 and ABI5 TFs (Pan et al., 2020). Upregulation of the expression of JA ZIM-domain protein indicates their possible role in the activation of ABI3 and ABI5 for the spatiotemporal safeguard seed germination process.

The role of WRKY TFs in response to coleoptile elongation in rice under submergence is not well studied yet. However, studies on WRKY TFs reported that it plays a role in hypoxic stress response in persimmon (Zhu et al., 2019), rice (Shiono et al., 2014; Mohanty et al., 2016), and sunflower (Raineri et al., 2015), and submergence tolerance in rice (Viana et al., 2018). Recent studies on WRKY stated that a regulatory module composed of WRKY33 and WRKY12 together with RAP2.2 plays a key role in hypoxia tolerance in *Arabidopsis* (Tang et al., 2021). During submergence stress, interaction of *WRKY 33* with *WRKY12* upregulates the expression of *RAP2.2*, which acts downstream of both *WRKY33* and *WRKY12*. RAP2.2 is an ethylene response TF that normally regulates genes associated with ethylene production, metabolism, and induction of genes encoding sugar metabolism and fermentation pathway enzymes (Hinz et al., 2010).

The identification of high enrichment of DRE-like elements associated with NAC TF in all groups of genotypes and expression of NAC genes suggest their role in regulating germination and coleoptile elongation together with other hormones to maintain membrane integrity. Similarly, the HD-ZIP (AtHB) gene acts downstream of P1F1 and regulates activation of hypocotyl cell wall composition and elongation through hormone-mediated development, particularly BR (Sorin et al., 2009; Capella et al., 2015). However, tolerant genotypes lack binding sites for BR-regulated TFs such as BES/BZR as well as bHLH (PIFs), whereas these are highly enriched particularly in highly tolerant and extremely tolerant genotypes (Tables 2, 3). In darkness, PIFs are normally active and involved in the regulation of gene expression to stimulate the skotomorphogenic response (Leivar et al., 2008). BZR1 and PIF4 interact with each other and regulate BR-induced gene expression (Martínez et al., 2018). There is also identification of ARR-B type binding site-like associated with ARR-B TFs. Type-B ARR binding sites are linked to the transcriptional regulation by TFs such as BZR2*;* BEH1, 2, 3, 4*;* BIM1*;* and MYB30 (Zubo and Schaller, 2020). MYB30 cooperates with BES1 and regulates the expression of BR-induced genes (Lee et al., 2009). Moreover, BR and GA are known to regulate cell elongation in rice, and BR regulates cell elongation by controlling GA metabolism (Tong et al., 2014). It seems that there is a cross-talk between BR, GA, and other hormones for the contribution of coleoptile elongation (Figure 2).

Interestingly, BBRE-element-like/G-box-like/E-box-like elements associated with TF BIM1, BIM3, and BEH1 are highly enriched in the extremely tolerant natural genotypes that have maximum coleoptile elongation (Figure 2 and Table 3). These genes may play a role in longer elongation of their coleoptiles compared to the highly tolerant genotypes. Besides the contribution of the BES1/BZR1 and PIF4 interaction for the longer elongation of coleoptiles in the extremely tolerant genotypes, BEE2 could also be involved in the BR signaling pathway for the maximum elongation of coleoptile in those two genotypes. Additionally, BAM8 (β-amylase–like proteins), a bHLH TF, could be acting as a metabolic sensor by interacting with the BR signaling pathway (Soyk et al., 2014).

Besides these binding sites, there were identifications of binding sites for TFs such as ABR1, HD-ZIP (ATHB4), TCP, ZNF, E2F, AS2 (LBD), NAC, and HSF (Tables 1-3, 5, 6). The roles of these TFs have not been studied in response to submergence stress. However, they could be involved in different protective roles during germination and coleoptile elongation in response to submergence such as HSF in protecting and preventing cellular responses (Liberek et al., 2008), ABR1TF in relation to mechanical stress, and NAC TF in controlling homeostasis of the cells. C2H2 zinc finger proteins could be involved in targeting the antioxidant genes associated with ROS scavenging (Han et al., 2020). TFs such as E2F and TCP could be involved in cell elongation and proliferation in a GA dependent pathway (Kieffer et al., 2011).

## Conclusion

Germination and coleoptile elongation of diverse rice genotypes vary in response to submergence. Promoter architecture of upregulated genes associated with different tolerant genotypes suggests a fine-tuning at the transcriptional level that affects the phenotype of different genotypes. The germination and elongation of rice coleoptiles in response to submergence tolerance in all three genotype groups could be due to a combination of mechanisms involving different TFs such as MYB, bZIP, AP2/ERF, ARF, WRKY, ZnF, MADS-box, NAC, AS2, DOF, E2F, ARR-B, and HSF, and hormonal regulation by GA, ABA, ethylene, JA, and auxin. There could be re-balance between GA and ABA to activate other TFs and stress-responsive genes for the elongation of coleoptile to escape the submergence stress. Interestingly, the longer coleoptile elongation in the highly tolerant and extremely tolerant genotypes could be due to the involvement of additional TFs such as bHLH and BES/BZR along with BR signaling and a cross-talk with other signaling pathways. Moreover, the maximum elongation in the two extremely tolerant natural genotypes might be due to the additional transcriptional regulatory mechanism governed by TFs such as BEE2, BIM1, BIM3, and BAM8, which are not present in the other two genotype groups. The variation in coleoptile elongation in different groups of genotypes is certainly due to the difference in transcriptional regulatory mechanism controlled by specific TFs along with a synergistic cross-talk interaction between different hormones, which needs further experimental validation. This analysis provides a potential mechanism of transcriptional regulation across rice genotypes from diverse backgrounds, which may be helpful for rice breeding targets for direct seeding to improve rice production.

## Data Availability Statement

The original contributions presented in the study are included in the article/Supplementary Material, further inquiries can be directed to the corresponding author/s.

## Author Contributions

BM designed and analyzed the data, and wrote the manuscript.

## Conflict of Interest

The author declares that the research was conducted in the absence of any commercial or financial relationships that could be construed as a potential conflict of interest.

## Footnotes

1 www.gene-regulation.com

2 http://www.dna.affrc.go.jp/htdocs/PLACE/

3 http://arabidopsis.med.ohio-state.edu/

4 http://PlantPAN.itps.ncku.edu.tw

5 http://rapdb.dna.affrc.go.jp/

## Abbreviations

ABA: Abscisic Acid
ABF1/2: ABA-responsive element binding factor 1/2
ABI3/4/5: Abscisic Acid-Insensitive 3/4/5
ABR1: AP2-like ABA repressor 1
ABRE: Abscisic Acid Response Element
AGL: Agamous-like
AP2/B3: APETALA2/B3
AP2/ERF: APETALA2/Ethylene-Responsive Element
ARR-B-Type-B Arabidopsis Response Regulator, ARF: Auxin Response Factor; ATHB4 Arabidopsis thaliana Homeobox 2
AUX1: Auxin influx carrier Auxin Resistant 1
BAM 8: BZR1-BAM 8
BAM1: β-amylase (BAM)–like 2
BEE: BR Enhanced Expression
BEH4: BE*S1/BZR1* H*omolog 4*
BES1: BRASSINOSTEROID INSENSITIVE1-ETHYL METHANESULFONATE-SUPPRESSOR 1
bHLH: basic Helix-Loop-Helix
BIM: BES1-Interacting MYC-Like
BIN2: Brassinosteroid Insensitive 2
BPC: Basic Penta Cysteine
BR: Brassinosteroid
bZIP: Basic Leucine Zipper
BZR1/2: Brassinazole-Resistant 1/2
CArG-box: ‘C-A rich-G-box
CAMTA: Calmodulin binding transcription factor
CBF: C-repeat Binding Factor
CCA1: circadian clock associated 1
C2H2: CCHH Zinc Finger
C3H: CCCH Zinc Finger
CCCH CPRF 5/6/7: Common Plant Regulatory Factor 5/6/7
cpm1: coleoptile photomorphogenesis 1
DOF: DNA-Binding with One Finger
DPBF-1/2: Dc3 Promoter Binding Factor 1/2
DREB: Dehydration-Responsive Element-Binding
E2F: E2 factor
EIL: Ethylene-Insensitive 3 (EIN3)-like protein3 (EIL3)
EIN: Ethylene-Insensitive 3
ERF: Ethylene Response Factor
GA: Gibberellic Acid
GARE: Gibberellic Acid Response Element
GBF: G-Box Binding Factor
HD: ZIP-Homeodomain Leucine Zipper
HSF: Heat Shock Factor
HY5: Elongated Hypocotyl 5
IAA: Indole − 3 − Acetic Acid
JA: Jasmonate
JA: Jasmonic Acid
JARE: Jasmonic acid Response Element
JAZ: Jasmonate ZIM domain
LBD: Asymmetric Leaves 2 (ASL2)/Lateral organ boundaries domain
MADS-box: Minichromosome Maintenance Factor 1 (MCM1 from Saccharomyces cerevisiae), Agamous (AG; from Arabidopsis thaliana), Deficiens (DEF: from Antirrhinum majus), and Serum Response Factor (SRF; from Homo sapiens)
MYB: Myeloblastosis
NAC, NAM: ATAF/, 2,and CUC2
NAM: No apical meristem
PIF1/3/4/7: Phytochrome Interacting Factor 1/3/4/7
RAP: Related to AP2
RAV: Related to ABI3/VP1
RNA: seq-RNA sequencing
ROS: Reactive oxygen species
SUB1: Submergence 1
SK1: SNORKEL1
SK2: SNORKEL2
TCP: TEOSINTE BRANCHED 1
TFs: Transcription Factors
TSS: Transcription Start Site
ZCT 1, 2, 3: Zinc Finger Catharanthus Transcription Factor 1,2,and 3
ZnF: Zinc Finger.
